# Pathogenesis and novel therapeutic perspectives in non-small cell lung cancer: Focusing on PI3K/AKT pathway regulation by traditional Chinese medicine monomers

**DOI:** 10.1097/MD.0000000000048775

**Published:** 2026-05-15

**Authors:** Shihu Gan, Shanjun Yang, Jipu Pan, Mingxing Wang

**Affiliations:** a The Second Affiliated Hospital of Heilongjiang University of Chinese Medicine, Harbin, Heilongjiang, China; b Heilongjiang University of Chinese Medicine, Harbin, Heilongjiang, China; c Shanghai TCM-Integrated Hospital, Shanghai University of TCM, Shanghai, China; d Shanghai University of Traditional Chinese Medicine, Shanghai, China.

**Keywords:** non-small cell lung cancer (NSCLC), PI3K/AKT signaling pathway, traditional Chinese medicine (TCM) monomers

## Abstract

Non-small cell lung cancer (NSCLC) remains a leading cause of global cancer mortality, with treatment efficacy limited by drug resistance and tumor heterogeneity. The phosphatidylinositol 3-kinase (PI3K)/AKT signaling pathway functions as a central “command hub” that integrates diverse oncogenic signals and coordinates downstream processes to drive tumor progression. Active monomers derived from traditional Chinese medicine (TCM) represent a promising therapeutic strategy due to their multi-target regulatory properties, yet a systematic and critical review of their mechanisms and translational potential is warranted. This is a comprehensive narrative review that synthesizes and critically evaluates the current body of literature on the role of the PI3K/AKT pathway in NSCLC pathogenesis and the potential of TCM monomers to modulate this pathway. The pathogenesis of NSCLC involves a dynamic, interconnected network of mechanisms, with PI3K/AKT as a core regulatory hub. TCM monomers modulate this pathway through distinct mechanisms: direct inhibition of core components (e.g., curcumol, fucoxanthin); modulation of upstream receptors (e.g., dictamnine, shikonin); regulation of micro-RNA networks (e.g., anwulignan, icariin); induction of oxidative stress (e.g., plumbagin, berberine); and coordinated regulation of mitochondrial function and epithelial–mesenchymal transition (e.g., vitexin, cardamonin). Substantial preclinical evidence supports their antitumor efficacy. However, clinical translation is consistently hindered by critical gaps, including relatively high effective concentrations in vitro, a near-universal lack of pharmacokinetic data, and undefined direct molecular targets. TCM monomers targeting the PI3K/AKT pathway offer a promising multi-target strategy for NSCLC adjuvant therapy. Future research must prioritize elucidating their direct targets, optimizing pharmacokinetic profiles, and rigorously assessing safety to bridge the translational gap. Development guided by a biomarker-driven precision medicine framework and exploration of rational combinations with conventional therapies are essential to advance these agents towards clinical application.

## 1. Introduction

Lung cancer, a malignancy characterized by persistently high global incidence and mortality rates, poses a significant threat to human life and quality of life. According to GLOBOCAN 2022 data, there were approximately 9.7 million cancer-related deaths worldwide in 2022, of which about 1.8 million (approximately 18.5%) were attributable to lung cancer, ranking it as the leading cause of cancer mortality. The incidence and mortality rates of lung cancer have shown a year-on-year increasing pattern. Globally, there were approximately 20 million new cancer cases in 2022, with lung cancer accounting for about 2.57 million new cases. This represents 12.85% of all new cancer diagnoses and 20.4% of all cancer deaths, solidifying its position as the most lethal malignancy.^[1]^ Compared to data from the International Agency for Research on Cancer’s GLOBOCAN 2020 database, which reported approximately 1.8 million lung cancer deaths (accounting for roughly 18% of the nearly 10 million total cancer deaths), the current proportion demonstrates a significant increase.^[2]^ Based on the microscopic appearance and morphology of malignant cells, lung cancer is histologically categorized into small cell lung cancer and non-small cell lung cancer (NSCLC). NSCLC constitutes over 80% of all lung cancer cases and primarily encompasses squamous cell carcinoma and adenocarcinoma, thereby representing a major focus of both research and therapeutic strategies.^[3]^

Modern medicine has achieved remarkable success in the treatment of NSCLC. From traditional surgical interventions to the optimization of radiotherapy and chemotherapy, alongside the precise application of targeted therapy and immunotherapy, and the maturation of multidisciplinary team approaches, these advancements have significantly prolonged patient survival and improved quality of life. Surgical resection remains the primary curative treatment option for patients with early-stage (Stage I–IIIA) NSCLC. The latest 20-year follow-up data from the International Early Lung Cancer Action Program confirms that patients with stage IA lung cancer diagnosed via low-dose computed tomography screening who undergo curative surgery achieve a 20-year lung cancer-specific survival rate of 86%. Notably, for those with pathological stage IA disease (tumor size ≤ 10 mm), this rate rises to 92%, representing a true clinical cure.^[4]^ Chemotherapy is one of the most conventional and widely used modalities in clinical oncology, administered either as a standalone treatment or in combination with surgery. Recent long-term follow-up studies have demonstrated that among patients with completely resected NSCLC, those receiving nivolumab in combination with chemotherapy achieved a 5-year overall survival (OS) rate of 65.4%, compared to 55.0% in the chemotherapy-alone group.^[5]^ Radiotherapy is a cornerstone of non-surgical cancer management. A phase II randomized study demonstrated that for patients with early-stage, node-negative NSCLC or isolated pulmonary parenchymal recurrence, the combination of stereotactic ablative radiotherapy with immunotherapy significantly improved the 4-year event-free survival rate from 53% to 77%.^[6]^ In the evolving landscape of treatment for resectable early-stage NSCLC, a 2015 Cochrane systematic review indicated that traditional platinum-based adjuvant chemotherapy provides an absolute improvement of 4% in the 5-year OS rate.^[7]^ With the advancement of immunotherapy, novel perioperative regimens combining immunotherapy with chemotherapy – as exemplified by the AEGEAN study – have demonstrated even greater benefits for patients with stage II–III disease. This approach reduces the risk of recurrence or death (event-free survival) by approximately 32% (HR = 0.68).^[8]^ Despite significant progress in NSCLC treatment, challenges such as drug resistance, tumor heterogeneity, and treatment-related toxicity persist. These limitations have prompted researchers to explore more precise intervention strategies at the molecular level. Among these, the phosphatidylinositol 3-kinase (PI3K)/AKT signaling pathway – a central hub regulating cell survival and proliferation – has emerged as a critical therapeutic target. Consequently, elucidating its pathogenic mechanisms and developing precision treatment strategies based on these findings are essential for further improving patient survival outcomes.

NSCLC is a malignancy driven by multiple genetic abnormalities. Its pathogenesis encompasses complex pathological processes, including uncontrolled proliferation mediated by the PI3K/AKT pathway, dysregulation of programmed cell death, epithelial–mesenchymal transition (EMT), angiogenesis, a chronic inflammatory microenvironment, and oxidative stress imbalance. In recent years, traditional Chinese medicine (TCM) has demonstrated multi-target regulatory potential in NSCLC treatment. Clinical evidence has accumulated supporting the role of TCM compound formulations in enhancing sensitivity to radiotherapy and chemotherapy, reducing toxic side effects, and improving quality of life. However, the complexity of compound ingredients hinders mechanistic elucidation, prompting a shift in research focus towards structurally defined TCM monomers. This comprehensive review aims to: summarize the pathogenesis of NSCLC; elucidate the core role of the PI3K/AKT signaling pathway in NSCLC malignant progression; and discuss strategies for developing TCM monomers based on pathway regulation. The objective is to provide new perspectives for precision therapy and drug development in NSCLC.

Therefore, elucidating the pathogenic mechanisms of NSCLC, particularly the role of the PI3K/AKT hub, and identifying therapeutically actionable biomarkers are the cornerstones for developing the next-generation of precision therapies, a paradigm that has already been revolutionized by biomarker-driven approaches in oncology, such as those targeting epidermal growth factor receptor (EGFR) mutations in lung cancer.^[9]^

## 2. Pathogenesis of NSCLC

### 2.1. Inflammatory response in NSCLC

Chronic inflammation is a well-established contributor to lung cancer progression. The inflammatory infiltrate within the tumor microenvironment (TME) comprises diverse populations of leukocytes, which are pivotal drivers of tumorigenesis through the secretion of a wide array of cytokines, chemokines, and cytotoxic mediators. This complex signaling network creates a pro-tumorigenic milieu that facilitates cancer cell survival, proliferation, and metastasis.^[10]^ The leukocyte infiltrate in tumors includes macrophages, neutrophils, lymphocytes, natural killer cells, dendritic cells, and mast cells.^[11]^ While some of these cells can antagonize tumor development, others actively promote it.

#### 2.1.1. Macrophages: dual roles and polarization

Macrophages, critical components of the immune system, play pivotal roles in tissue homeostasis, pathogen defense, and inflammatory mediation.^[12]^ They exhibit functional plasticity: capable of releasing inflammatory factors to mount immune responses and facilitate tissue repair, but also of acquiring pro-tumorigenic functions that promote cancer progression within the TME.^[13]^ During early tumorigenesis, cancer cells recruit tumor-associated macrophages.^[14]^ Tumor-associated macrophages are broadly classified into M1 and M2 subtypes.^[15]^ M1 macrophages, typically activated by Toll-like receptor ligands or Th1 cytokines, can inhibit tumor progression. Conversely, M2 macrophages, polarized by Th2 cytokines, secrete anti-inflammatory cytokines and contribute to an immunosuppressive microenvironment conducive to tumor development.^[16]^

#### 2.1.2. Tumor-associated neutrophils

Similar to macrophages, neutrophils in the TME are categorized into antitumor N1 and pro-tumorigenic N2 subtypes.^[17]^ The pro-tumorigenic functions of N2 tumor-associated neutrophils (TANs) include releasing reactive oxygen species (ROS) to induce DNA damage and promote mutagenesis,^[18]^ as well as secreting matrix metalloproteinase-9 (MMP-9) to facilitate angiogenesis and support tumor growth.^[19]^

#### 2.1.3. Pro-tumorigenic inflammatory cytokines

Cytokines secreted by immune cells are critical for tumor initiation and progression.

Tumor necrosis factor-alpha, primarily secreted by macrophages, is a central inflammatory mediator.^[20]^ It promotes tumor development by inducing EMT in cancer cells, thereby enhancing their migratory and invasive capabilities.^[21]^

Interleukin-8 is a frequently overexpressed pro-inflammatory cytokine in malignancies. In lung cancer, interleukin-8 stimulates cancer cells, enhancing protein O-GlcNAcylation.^[22]^ This post-translational modification helps tumor cells adapt to a challenging TME characterized by hypoxia and nutrient deprivation.^[23]^

Interleukin-6 promotes carcinogenesis and the proliferation of lung cancer stem cells by upregulating DNA methyltransferase 1. This upregulation is accompanied by the downregulation of tumor suppressors p53 and p21 due to DNA hypermethylation.^[24]^

Moreover, inflammation supplies the TME with critical molecular components, including growth factors that sustain proliferative signaling, survival factors that inhibit apoptosis, pro-angiogenic factors, and extracellular matrix (ECM)-modifying enzymes that promote invasion and metastasis.^[25]^ Collectively, these processes underscore the significant contribution of the inflammatory response to NSCLC development and progression.

### 2.2. EMT in NSCLC progression

EMT is a physiological process essential for normal embryonic development, but it also occurs in response to tissue injury, wound healing, and organ fibrosis.^[26]^ Numerous in vitro and vivo studies have demonstrated that EMT plays a central role in tumorigenesis, as well as in cancer invasion and metastasis.^[27]^ During EMT, epithelial cells progressively lose their epithelial characteristics (e.g., cell–cell adhesion, apico-basal polarity) and acquire mesenchymal traits, including alterations in cell shape and significant cytoskeletal reorganization. Consequently, cells gain enhanced invasive and migratory capabilities, enabling them to invade the surrounding stroma and disseminate to distant organs via the bloodstream and lymphatic systems.^[27,28]^

Cancer stem cells, which possess the ability to differentiate into various cell types within a tumor and drive tumor initiation, progression, and recurrence, often undergo EMT during their differentiation into cancer cells.^[29]^ The EMT process can be initiated by signaling pathways such as transforming growth factor-beta (TGF-β). Upon ligand-induced receptor activation, TGF-β initiates the SMAD pathway, leading to the phosphorylation of SMAD2 and SMAD3. These then form a complex with SMAD4 and translocate to the nucleus. In lung adenocarcinoma cells, this complex activates the long non-coding RNA lncRNA1, triggering the EMT program and enhancing cell migration and invasion.^[30,31]^

The upregulation of EMT transcription factors, such as Snail 1, drives the transition of cancer cells from a differentiated epithelial state to a stem cell-like state, thereby enhancing their metastatic potential.^[32]^ Elevated expression of Snail 1 is associated with enhanced invasive and metastatic potential in cancers such as lung cancer. Conversely, studies have shown that reducing Snail 1 expression can improve the efficacy of various chemotherapeutic and immunotherapeutic agents.^[33,34]^ Furthermore, keratin 6A, a type II cytokeratin, promotes the proliferation and metastasis of lung cancer cells in lung adenocarcinoma by facilitating the EMT process.^[35]^

On the other hand, EMT can confer cancer stem cell-like properties upon lung cancer cells. These cells possess self-renewal and differentiation capacities, enabling them to initiate new tumors in vivo and exhibit resistance to multiple therapeutic modalities, thereby contributing to tumor growth, metastasis, and recurrence.^[36]^ Consequently, EMT plays a critical role in the progression and metastatic dissemination of lung cancer.

### 2.3. Oxidative stress in NSCLC pathogenesis

ROS are oxygen-derived molecules generated during aerobic metabolism. Under normal physiological conditions, cells maintain a basal level of ROS that participates in cellular signaling and metabolic regulation. When redox homeostasis is disrupted, however, ROS levels become abnormally elevated, leading to a state of oxidative stress.^[37]^

#### 2.3.1. Sources and consequences of oxidative stress

The primary enzyme responsible for generating ROS is NADPH oxidase, a membrane-bound multi-component enzyme complex present in both phagocytic and non-phagocytic cells.^[38]^ Oxidative stress can induce DNA damage, genomic instability, and alterations in key signaling pathways, thereby contributing directly to tumor initiation and progression.^[39]^ ROS-induced oxidative DNA damage is a key driver of carcinogenesis, encompassing strand breaks, base modifications, and replication errors that collectively promote genomic instability and cancer cell proliferation.^[40]^ Beyond direct DNA damage, oxidative stress modulates the TME by promoting inflammation and angiogenesis, and by affecting antitumor immune responses.^[39]^

#### 2.3.2. Oxidative stress and therapy resistance

Oxidative stress not only promotes the proliferation and survival of tumor cells but also contributes to the development of therapy resistance.^[41]^ Cancer cells can activate adaptive antioxidant pathways to survive under high ROS conditions. For instance, the antioxidant enzyme glutathione peroxidase 4 maintains membrane integrity by detoxifying lipid peroxides.^[42]^ Epigenetic dysregulation can lead to aberrant activation of such antioxidant pathways, enabling immune evasion and conferring resistance to therapies.^[43]^

#### 2.3.3. Impact on the immune microenvironment

Oxidative stress exerts a profound influence on the immune landscape of tumors. It can impair the function of immune effector cells, enabling cancer cells to evade immune recognition and attack.^[44]^ A critical example is the effect on CD8 + T cells, which are crucial for controlling tumor growth. Excessive oxidative stress within the TME can lead to CD8 + T cell functional exhaustion, resulting in immune escape and promoting carcinogenesis.^[45]^

#### 2.3.4. The dual role of oxidative stress

Notably, oxidative stress exhibits a context-dependent dual role in the TME. On one hand, it can promote tumor progression by inducing cancer cells to release factors like lactate and ROS, thereby remodeling the microenvironment to support growth. On the other hand, excessive or sustained oxidative stress can trigger apoptosis and immunogenic cell death, thereby inhibiting tumor development.^[46]^ This duality highlights the complexity of targeting redox balance for cancer therapy.

### 2.4. Autophagy in NSCLC pathogenesis

Autophagy is a conserved catabolic process in which cytoplasmic components are sequestered within autophagosomes and delivered to lysosomes for degradation, thereby maintaining cellular homeostasis and facilitating recycling.^[47]^ Multiple forms of selective autophagy have been identified, including mitophagy,^[48]^ pexophagy,^[49]^ ER-phagy,^[50]^ and nucleophagy.^[51]^ These selective subtypes can induce programmed cell death, thereby suppressing early tumorigenesis.^[52]^

#### 2.4.1. Mechanisms of autophagic dysregulation in NSCLC

Dysregulation of autophagy, manifesting as either inhibition or excessive activation, plays a complex role in NSCLC pathogenesis.

Excessive activation: hyperactivation of autophagy can be triggered by multiple mechanisms. Under nutrient deprivation, 5’-AMP-activated protein kinase (AMPK) – a key cellular energy sensor – is activated. Activated AMPK phosphorylates and activates TSC2 (an inhibitor of mechanistic target of rapamycin [mTORC1]) and can directly phosphorylate the mTORC1 subunit Raptor, collectively suppressing mTORC1 activity and inducing autophagy.^[53]^ Furthermore, oncogenic KRAS activates the P38 pathway to enhance ULK1 activity. ULK1 then phosphorylates PI4KB to generate PI4P, which recruits complexes that facilitate ATG8ylation and the formation of non-canonical autophagosomes, leading to excessive autophagy.^[54]^

Inhibition of autophagy: several key pathways suppress autophagy. Mammalian target of rapamycin (mTOR) is a central autophagy inhibitor^[55]^ that suppresses autophagosome formation and restricts lysosomal biogenesis and function.^[56]^ Overexpression of EGFR activates the Ras–Raf–MEK–extracellular signal-regulated kinase (ERK) pathway to promote survival and proliferation. Concurrently, activated Ras binds and activates PI3K, leading to Akt–mTORC1 axis activation, which enhances protein synthesis while inhibiting autophagy.^[57]^ Additionally, mitochondrial STAT3 can suppress oxidative stress-induced autophagy, thereby protecting mitochondria from mitophagy.^[58]^ Beclin-1, a key regulator that induces autophagic vesicle initiation and expansion,^[59]^ is frequently dysregulated in cancer.

#### 2.4.2. The dual role of autophagy in cancer progression

The role of autophagy in cancer is context-dependent, exerting neutral, tumor-suppressive, or tumor-promoting effects at different disease stages.

Tumor-suppressive role: In early tumorigenesis, autophagy acts as a tumor suppressor by clearing damaged organelles and protein aggregates (e.g., oncogenic p62), thereby mitigating harmful stimuli and inhibiting tumor initiation, proliferation, invasion, and metastasis.^[60]^ Supporting this, heterozygous deletion of the *Beclin 1* gene, which reduces autophagic activity, increases the incidence of spontaneous malignancies, including lung cancer.^[61]^ Conversely, inhibition of autophagy can lead to tissue damage, necrosis, chronic inflammation, and genomic instability, altering the TME and increasing cancer risk.^[60]^

Tumor-promoting role: In established tumors, excessive autophagy can support rapid cancer cell proliferation by providing substantial energy and nutrients through catabolism.^[62]^ Moreover, under hypoxic conditions, hyperactivated autophagy can selectively degrade connexin-43, impairing natural killer cell-mediated cytotoxicity and facilitating immune evasion.^[63]^

Therefore, autophagy exhibits a dual role in NSCLC development, with its function being critically dependent on the specific stage and context of disease progression.

### 2.5. MMPs in NSCLC pathogenesis

Matrix metalloproteinases (MMPs) are a family of Zn^2+^-dependent endopeptidases that play critical roles in cancer progression by degrading components of the ECM and basement membrane (BM). They are produced by tumor cells, surrounding stromal fibroblasts, and tumor-associated inflammatory cells.^[64,65]^ Through the proteolytic cleavage of various ECM and BM substrates, MMPs facilitate extensive tissue remodeling, thereby creating a permissive microenvironment conducive to cancer cell invasion, angiogenesis, and metastasis.^[66]^

#### 2.5.1. Key MMPs and their functions in NSCL

Specific members of the MMP family have been extensively studied for their distinct contributions to NSCLC pathogenesis.

MMP-9 (gelatinase B): As one of the largest proteolytic enzymes in the MMP family, MMP-9 enhances the ability of tumor cells to breach the BM, promoting invasion and metastasis. Elevated serum levels of MMP-9 in NSCLC patients are strongly correlated with poorer 3-year survival rates.^[67]^ Its expression is closely linked to tumor differentiation status, being significantly higher in undifferentiated tumors.^[68]^ Mechanistically, MMP-9 exerts a potent pro-angiogenic function by catalyzing the release of vascular endothelial growth factor (VEGF) from the tumor stroma, thereby regulating new blood vessel formation that supports tumor growth and metastasis.^[69,70]^

MMP-2 (gelatinase A): The MMP-2 gene, located on human chromosome 16q21, specifically degrades type IV collagen in the BM and other matrix components. Given the ECM’s role as a physical barrier to metastasis, high MMP-2 expression in tumor cells accelerates matrix degradation, thereby promoting tumor invasion and metastasis.^[71]^ Experimental evidence indicates that the removal of MMP-2 can delay tumor growth, further substantiating its tumor-promoting role.^[69]^

MMP-14 (MT1-MMP): This membrane-anchored MMP plays a critical role by directly degrading ECM/BM proteins and, importantly, by activating the pro-forms of MMP-2 and MMP-9, thereby amplifying the proteolytic cascade and enhancing cancer cell migration.^[72]^ Furthermore, MMP-14 can stimulate the EGFR signaling pathway by mediating the proteolytic processing and activation of heparin-binding epidermal growth factor, thereby increasing cell proliferation and tumor growth.^[73]^

MMP-28 (epilysin): Originally cloned from human keratinocyte and testis cDNA libraries, MMP-28 can induce EMT and enhance cell invasion via a TGFβ-dependent mechanism, thereby promoting lung cancer development.^[74]^

MMP-11 (stromelysin-3): MMP-11 exhibits a context-dependent dual role: it can accelerate cancer progression by inhibiting apoptosis and promoting migration, yet it may also exert anticancer effects by suppressing metastasis in certain contexts.^[75]^

In summary, MMPs are pivotal mediators of NSCLC progression through their coordinated actions in ECM degradation, angiogenesis induction, growth factor activation, and EMT promotion, making them potential therapeutic targets.

### 2.6. The gut–lung axis in NSCLC pathogenesis

Significant bidirectional crosstalk exists between the gastrointestinal and respiratory tracts, a phenomenon termed the gut–lung axis. This axis connects the intestinal and pulmonary niches via the circulatory system, facilitating interactions between microbial communities and immune functions to achieve mutual regulation.^[76,77]^ The human gut harbors a vast and diverse microbial community – the gut microbiota – comprising approximately 10^13^ to 10^14^ microorganisms and over 1000 distinct bacterial species.^[78]^ Its composition evolves from birth, stabilizing into a relatively balanced state in adulthood, with a ‘core’ microbiome shared among most healthy individuals that includes genera such as *Bifidobacterium*, *Eubacterium*, *Bacteroides*, *Faecalibacterium*, and *Ruminococcus*.^[79,80]^ The gut microbiota and the host maintain a mutually beneficial symbiotic relationship through the metabolism–immune–neuroendocrine network.^[81]^

#### 2.6.1. Mechanisms of gut microbiota impact on NSCLC

The gut microbiota plays a significant role in the initiation and progression of lung cancer, with dysbiosis directly or indirectly associated with carcinogenesis.^[82]^ Several key mechanisms have been elucidated:

Immunomodulation via microbial components: specific gut bacteria can systemically influence antitumor immunity. For instance, extracellular vesicles derived from *Bifidobacterium* can traverse the gut barrier, enter circulation, and be taken up by lung tumor cells. This uptake triggers signaling pathways that upregulate programmed death-ligand 1 (PD-L1) expression and activate local immunity, thereby enhancing CD8^+^ T cell-mediated tumor killing.^[83,84]^

Systemic inflammation and metabolite-mediated effects: gut dysbiosis can compromise the intestinal mucosal barrier, allowing bacteria and their metabolites to enter the bloodstream and modulate systemic inflammatory and immune responses. Bacterial antigens can activate Toll-like receptor 4 in intestinal immune cells, elevating circulating interleukin-1β (IL-1β) levels and activating the nuclear factor kappa B (NF-Κb) pathway to induce a pro-tumorigenic pulmonary inflammation^[84]^. Furthermore, elevated plasma levels of the microbial metabolite 12,13-DiHOME can reduce pulmonary regulatory T cell numbers, diminishing anti-inflammatory effects and promoting lung cancer progression. Certain gut bacteria can also convert primary bile acids into secondary metabolites like deoxycholic acid and lithocholic acid, which are capable of causing DNA damage and further driving carcinogenesis.^[85]^

#### 2.6.2. Clinical evidence linking gut microbiota to NSCLC

Numerous clinical studies support the critical role of gut microbiota composition in NSCLC prognosis and therapy response.

Beneficial taxa: the presence of specific bacterial species is associated with improved outcomes. For example, patients harboring *Akkermansia muciniphila* exhibit higher objective response rates and longer median OS.^[86]^ *Peptococcus* species appear to confer protection against NSCLC via CD45-mediated mechanisms on HLA-DR^+^CD4^+^ T cells.^[87]^ *Clostridium butyricum* therapy has been shown to enhance the efficacy of immune checkpoint blockade in lung cancer.^[88]^

Dysbiosis in NSCLC patients: comparative analyses reveal significant alterations in the gut microbiota of NSCLC patients versus healthy controls. Patients show higher fecal abundances of *Rikenellaceae*, *Prevotella*, *Streptococcus*, *Lactobacillus*, *Bacteroides*, *Oscillospira*, and *Enterobacteriaceae*, while *Micrococcaceae*, *Dialister*, and *Sutterella* are less abundant.^[89,90]^ Notably, levels of beneficial, butyrate-producing bacteria (e.g., *Faecalibacterium prausnitzii*, *Ruminococcus*, and *Clostridium* cluster I) are significantly reduced.^[91]^ Conversely, high levels of *Bacillus*and *Akkermansia muciniphila* have also been implicated in promoting lung carcinogenesis in some contexts, highlighting the complexity of these interactions.^[92]^

Collectively, these findings underscore the gut–lung axis as a critical regulatory circuit in humans. It plays a significant role not only in the pathogenesis of lung cancer but also in modulating systemic inflammation and antitumor immune responses, presenting a novel avenue for therapeutic intervention.

### 2.7. Pyroptosis in NSCLC pathogenesis

Pyroptosis is an inflammatory form of programmed cell death characterized by distinct morphological features, including cell swelling, loss of plasma membrane integrity, formation of pyroptotic bodies, and the release of inflammatory cytokines.^[93]^ Recent advances indicate that pyroptosis is closely associated with the pathogenesis, progression, prognosis, and drug resistance in lung cancer.^[94]^

#### 2.7.1. Molecular pathways of pyroptosis

Pyroptosis primarily occurs via 2 distinct pathways: canonical and non-canonical.^[95]^

The canonical pathway: this pathway depends on inflammasome activation and caspase-1.^[96]^ It is initiated when pattern recognition receptors recognize pathogen-associated molecular patterns or damage-associated molecular patterns. Following recognition, pattern recognition receptors recruit adaptor proteins and procaspase-1 to assemble a multiprotein complex known as the inflammasome (e.g., the NLRP3 inflammasome).^[97]^ Active caspase-1 then cleaves pro-interleukin-1β (pro-IL-1β) and pro-IL-18 into their mature, active forms. Crucially, it also cleaves gasdermin D (GSDMD), releasing its N-terminal fragment (GSDMD-NT) that oligomerizes to form pores in the plasma membrane, leading to cell lysis and inflammation.^[98]^

The non-canonical pathway: this pathway primarily relies on the activation of caspase-4, caspase-5 (in humans), or caspase-11 (in mice).^[99]^ Cytosolic lipopolysaccharide from gram-negative bacteria directly binds to and induces the auto-proteolytic activation of these caspases.^[100]^ The activated caspases then directly cleave GSDMD. Similar to the canonical pathway, the released GSDMD-NT forms membrane pores, causing cell swelling, rupture, and inflammatory content release.^[101]^ Concurrently, this pathway can indirectly activate the NLRP3 inflammasome, further promoting the maturation and release of IL-1β and amplifying the inflammatory response.^[102]^

#### 2.7.2. The dual role of pyroptosis in NSCLC

Emerging evidence indicates that pyroptosis exerts a context-dependent dual role in tumor biology.^[94,103,104]^

Tumor-suppressive effects: pyroptosis can directly eliminate cancer cells. It can inhibit pyruvate kinase M2, activate caspase-8 and caspase-3, and cleave gasdermin E, leading to the release of inflammatory factors and induction of pyroptosis in tumor cells.^[103]^ Alternatively, via the canonical pathway, activation of caspase-1 and subsequent GSDMD cleavage can also induce pyroptotic death in cancer cells.^[104]^

Tumor-promoting effects: conversely, pyroptosis can paradoxically fuel tumorigenesis. When pyroptotic cells release large amounts of pro-inflammatory cytokines (e.g., IL-1β, IL-18) and damage-associated molecular patterns, these substances persistently stimulate surrounding tissues, creating a chronic inflammatory microenvironment conducive to cancer development.^[105]^ The released cytokines can bind to receptors on tumor cell surfaces,^[106]^ promoting the expression of cell cycle-related genes. This accelerates the G1-to-S phase transition, driving proliferation, while simultaneously inhibiting apoptosis-related genes and reducing cell death.^[107]^ Furthermore, pyroptosis has been implicated in promoting tumor angiogenesis and stimulating the proliferation and metastasis of lung cancer cells.^[94]^

Thus, pyroptosis plays a complex and dual role in NSCLC development. It can exert antitumor effects by directly inducing cancer cell death, yet it may also promote tumor progression by fostering a chronic inflammatory and proliferative microenvironment.^[94,108]^

### 2.8. TME in NSCLC pathogenesis

The TME is a dynamic and complex ecosystem composed of tumor cells, diverse stromal cells, the ECM, and a myriad of soluble factors.^[109]^ The aberrant behavior of cancer cells – characterized by dysregulated proliferation, metabolism, and phenotype – actively reshapes the TME. This remodeling, including dysregulated glucose metabolism, lactate accumulation, and amino acid deprivation, fosters an immunosuppressive milieu conducive to tumor progression.^[110]^

#### 2.8.1. Stromal and immune components of the TME

The nonmalignant cellular components of the TME play critical and often paradoxical roles in NSCLC pathogenesis.

##### Cancer-associated fibroblasts (CAFs)

CAFs are a major stromal component that secrete abundant ECM proteins, such as collagen and fibronectin. These components provide both physical scaffolding for tumor cell attachment and growth,^[111]^ and biological signaling by engaging adhesion receptors to activate pro-tumorigenic intracellular pathways.^[112]^ Furthermore, the CAF-derived ECM can bind, sequester, and release growth factors, dynamically modulating tumor cell behavior.^[113]^

##### Myeloid-derived suppressor cells (MDSCs)

As key immunosuppressive cells, MDSCs establish an immunosuppressive milieu by producing metabolites such as ROS and nitric oxide, which directly inhibit the antitumor functions of T cells and natural killer (NK) cells.^[114,115]^ Beyond immune suppression, MDSCs also promote tumor progression by facilitating the formation of pre-metastatic niches that support neovascularization and tumor cell growth.^[116]^

##### TANs

TANs secrete a plethora of growth factors, chemokines, and cytokines that directly promote carcinogenesis and enhance tumor invasiveness.^[117]^

##### Dendritic cells

In contrast to immunosuppressive cells, dendritic cells, particularly the cDC1 subset, are pivotal for initiating antitumor immunity. They recognize danger signals, capture tumor antigens, and perform cross-presentation to activate tumor-specific CD8^+^ T cell responses.^[118,119]^

##### NK cells

The role of NK cells cells is context-dependent. While traditionally viewed as antitumor effectors, oncogenic cells can sometimes overexpress ligands for NK activation receptors, leading to a paradoxical state of NK cell activation that may, under certain conditions, inadvertently promote tumor progression.^[120]^

#### 2.8.2. Integrated role of the TME in NSCLC progression

The coordinated interplay of these components enables the TME to perform multifaceted functions critical for all stages of NSCLC development.^[121]^

##### Promoting proliferation and survival

The TME provides structural support, growth signals, and a nutrient-reprogrammed milieu that sustains NSCLC cell proliferation and survival.^[122]^

##### Enabling immune evasion

Through the actions of immunosuppressive cells (e.g., MDSCs) and metabolic alterations, the TME creates a shield that allows cancer cells to evade immune detection and destruction.^[122]^

##### Inducing angiogenesis

The TME secretes pro-angiogenic factors to stimulate the formation of new blood vessels, ensuring the supply of oxygen and nutrients necessary for tumor growth.^[123]^

##### Facilitating invasion and metastasis

By remodeling the ECM, enhancing cell motility, and establishing pre-metastatic niches, the TME critically enhances the invasive and metastatic potential of cancer cells, enabling their dissemination to distant organs.^[124]^

Thus, the TME is not a passive bystander but an active and indispensable regulator that orchestrates NSCLC progression from early tumorigenesis to advanced metastasis.

### 2.9. Angiogenesis in NSCLC pathogenesis

Tumor development is highly dependent on angiogenesis – the formation of new blood vessels from preexisting ones. This process provides essential oxygen, nutrients, and waste removal pathways for tumor growth and metastasis, making it a critical step in cancer progression.^[125]^

#### 2.9.1. Key molecular mediators and their mechanisms

Angiogenesis is a tightly regulated, multi-step process primarily driven by pro-angiogenic factors secreted by tumor and stromal cells.

##### VEGF

VEGF is the most critical pro-angiogenic factor.^[126]^ It primarily binds to vascular endothelial growth factor receptor (VEGFR)-2 on endothelial cells, inducing receptor dimerization, autophosphorylation, and downstream signaling activation.^[127]^ VEGF promotes angiogenesis through multiple concerted mechanisms: inducing endothelial cell proliferation by upregulating cell cycle proteins like cyclin D1^[128]^; enhancing endothelial migration via actin assembly and focal adhesion kinase activation^[129,130]^; remodeling the ECM by inducing MMP secretion to degrade the BM^[131]^; inhibiting endothelial apoptosis to stabilize nascent vasculature^[132,133]^; and increasing vascular permeability by stimulating nitric oxide and prostacyclin production. This heightened permeability facilitates plasma protein extravasation, providing a provisional matrix for vascular growth, and creates favorable conditions for tumor cell invasion and metastasis.^[134]^

##### Fibroblast growth factor (FGF)

The FGF family represents another important class of pro-angiogenic factors. FGF-1 induces endothelial cell activation, including detachment, migration, proliferation, and differentiation.^[135]^ FGF-2 regulates the splicing of VEGFR1 pre-mRNA, thereby promoting endothelial cell proliferation, survival, and vascular sprouting.^[136]^

#### 2.9.2. The sequential process of tumor angiogenesis

Following receptor activation by factors like VEGF and FGF, quiescent endothelial cells are converted into an activated state, characterized by the upregulation of integrins and MMPs.^[127]^ The angiogenic cascade then proceeds through defined stages:

##### Sprouting

Activated endothelial cells migrate directionally toward the tumor, proliferate, and form initial “vascular sprouts.”^[137]^

##### Lumen formation and pruning

Through complex intercellular interactions, these sprouts undergo lumenization and interconnect to form primitive capillary networks.^[137,138]^

##### Maturation and stabilization

The final steps involve the synthesis of a new BM and the recruitment of pericytes, which envelop the endothelial cells to form structurally stable, functional neovessels.^[138]^

#### 2.9.3. The hypoxic microenvironment and pathological angiogenesis

Hypoxia is a central driver of tumor angiogenesis.^[139]^ Within rapidly growing tumors, the hypoxic microenvironment induces the differentiation of cancer stem cells into endothelial-like cells, directly contributing to neovascularization.^[140]^ More importantly, hypoxia promotes “pathological angiogenesis,” resulting in the formation of numerous structurally and functionally aberrant vessels.^[140]^ These vessels are characterized by irregular lumens, high permeability, and disorganized branching, which severely compromise their transport function. This exacerbates intratumoral ischemia and hypoxia, establishing a vicious cycle. Hypoxia continuously activates hypoxia-inducible factor-1α (HIF-1α), which upregulates the expression of multiple pro-angiogenic factors (including VEGF), driving the formation of more chaotic vasculature.^[141]^

#### 2.9.4. Angiogenesis in NSCLC: specific drivers and clinical relevance

Angiogenesis plays a particularly critical role in NSCLC.^[142]^ Specific molecular axes have been identified as key promoters. For instance, the transcription factor SOX4, in conjunction with β-catenin, forms the SOX4/β-catenin axis to promote the self-renewal of lung cancer cells, thereby driving tumor development.^[143]^ Furthermore, the VEGF family promotes VEGF-A secretion via the SOX4–BMI1 axis, directly accelerating angiogenesis in NSCLC and facilitating more rapid disease progression.^[144]^

This continuous, dysregulated nutrient supply via pathological vessels is a hallmark of NSCLC and a major therapeutic target.

### 2.10. Immune regulation in NSCLC pathogenesis

Tumorigenesis is intrinsically linked to immune function. Under normal conditions, the immune system can recognize, monitor, and eliminate most malignant cells. However, a small subset of cancer cells evades this surveillance, leading to an ‘equilibrium’ phase where the immune system and cancer cells co-exist without clinical symptoms – a phenomenon termed immune escape.^[145]^

#### 2.10.1. Mechanisms of tumor immune escape

Tumor immune escape refers to the ability of cancer cells to avoid recognition and destruction by the immune system through various mechanisms, enabling their survival and proliferation.^[146]^ These mechanisms are multifaceted, including loss of tumor antigens, induction of immune suppression by tumor cells, absence of co-stimulatory signals on the tumor cell surface, and intrinsic anti-apoptotic effects.^[147–149]^ Specific molecular pathways driving immune escape in NSCLC include:

##### Hypoxia-induced pathways

Most tumor tissues exist in a hypoxic state, leading to the overexpression of hypoxia-inducible factors (HIF-1α and HIF-2α). This contributes significantly to tumor immune escape and promotes tumorigenesis.^[150]^

##### Neuroendocrine–immune axis activation

Tumor cells can stimulate neurons in the paraventricular nucleus of the hypothalamus in tumor-bearing mice, promoting pituitary production of α-melanocyte-stimulating hormone. This enhances myelopoiesis, leading to the accumulation of immunosuppressive tumor-associated myeloid cells. Elevated serum α-melanocyte-stimulating hormone levels in NSCLC patients positively correlate with peripheral myeloid cell proportions, confirming this pathway’s role in suppressing antitumor immunity and fostering tumor progression.^[151]^

##### Checkpoint modulation via metabolic and transcriptional regulation

Inhibition of the adenosine ADORA1–cAMP signaling axis modulates the expression of the transcription factor activating transcription factor 3 in cancer cells, leading to upregulation of PD-L1. This promotes the exhaustion of cytotoxic CD8^+^ T cells, facilitating immune escape.^[152]^

##### Co-stimulatory signal dysregulation

High expression of CD40 on tumor cells can bind to its ligand (CD40L), paradoxically leading to the activation of immunosuppressive T cell populations, thereby facilitating immune escape.^[153]^

#### 2.10.2. Evasion of apoptosis: the role of ELF3

A key strategy for immune evasion is resisting programmed cell death. E74-like ETS transcription factor 3 (ELF3) is one of the most upregulated genes in advanced-stage NSCLC cells. It regulates the cell cycle and proliferation, but critically, it also inhibits the activity of pro-apoptotic proteins. ELF3 binds to and activates the promoter regions of anti-apoptotic genes while suppressing the transcription of pro-apoptotic genes, thereby attenuating apoptotic signaling in tumor cells and enabling their survival despite immune pressure.^[154]^

In summary, NSCLC employs a diverse arsenal of strategies – spanning hypoxia response, neuroendocrine manipulation, metabolic reprogramming, checkpoint expression, and intrinsic apoptosis resistance – to subvert immune surveillance. This multifaceted immune dysregulation is a cornerstone of NSCLC pathogenesis and a major target for therapeutic intervention.

**Figure 1.**
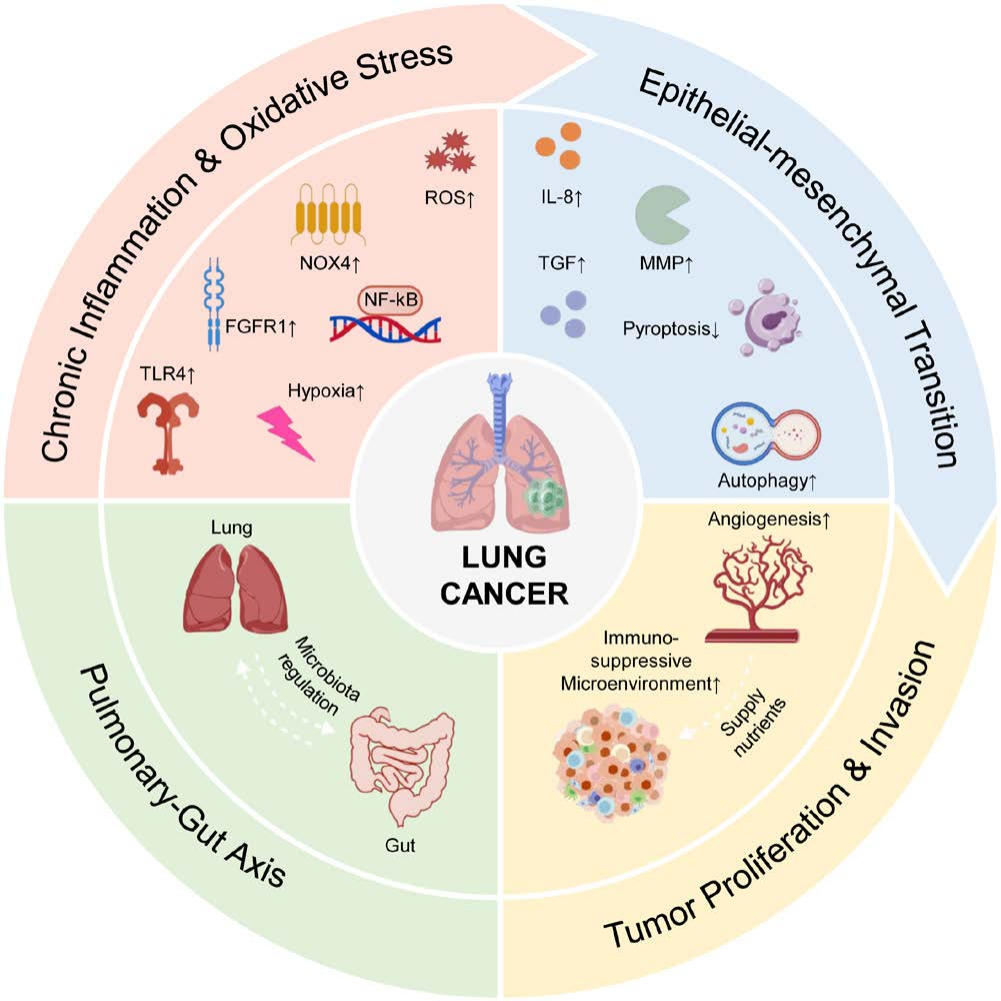
The pathogenesis of NSCLC is not governed by isolated events but by a dynamic, interconnected network of molecular, and cellular mechanisms. NSCLC = non-small cell lung cancer.

In summary, as summarized in Figure 1, the pathogenesis of NSCLC is not governed by isolated events but by a dynamic, interconnected network of molecular and cellular mechanisms. Chronic inflammation and oxidative stress, as core drivers, mutually reinforce each other to establish a pro-carcinogenic foundation.^[155]^ They primarily function within the TME, which acts as a central hub integrating diverse signaling pathways. By fostering a hypoxic, acidic, and immunosuppressive niche, the TME further exacerbates inflammation and oxidative stress, creating a self-sustaining vicious cycle.^[156]^ Within this permissive milieu, EMT serves as the core program through which cancer cells acquire invasive and metastatic capabilities.^[157]^ Autophagy and pyroptosis play context-dependent dual roles in cell fate decisions, with their ultimate outcomes dictated by microenvironmental cues.^[158]^ MMPs, acting as “remodelers” of the ECM, clear the path for invasion and metastasis.^[159]^ The collective output of these intertwined processes gives rise to and is sustained by 2 major supportive pillars: tumor angiogenesis for nutrient supply and the formation of an immunosuppressive microenvironment to facilitate immune escape.^[160]^ Furthermore, the emerging gut–lung axis reveals that the gut microbiota acts as a systemic modulator, deeply involved in constructing and regulating this local network by modulating systemic immunity and inflammation.^[161]^ Consequently, effective therapeutic strategies must focus on deconstructing multiple key nodes within this interactive network, rather than targeting isolated pathways (Fig. 1).

## 3. PI3K/AKT pathway: core components and their pathogenic contributions to non-small cell lung carcinogenesis

### 3.1. Structural composition and activation of the PI3K/AKT pathway

#### 3.1.1. Core components: PI3K and AKT

PI3K is an evolutionarily conserved intracellular lipid kinase, classified into 3 major types (I, II, and III) based on substrate specificity and sequence homology.

Class I PI3K is the most extensively studied due to its critical roles in cell proliferation and survival, and is further subdivided into IA and IB subfamilies according to receptor coupling.^[162]^

Class IA PI3K exists as a heterodimer composed of a catalytic subunit (p110α, p110β, or p110δ) and a regulatory subunit (p85α, p85β, or p85γ). Its activation can be mediated through G protein-coupled receptors, the small GTPase RAS, or receptor tyrosine kinases (RTKs), and it exhibits dual functionality as both a lipid kinase and a serine/threonine protein kinase.^[163,164]^

Class IB PI3Ks are exclusively activated by G protein-coupled receptors,^[164]^ mediating signal transduction and immunomodulation with critical roles in inflammatory responses and immune cell activation.^[165]^

Protein kinase B (PKB/AKT), a serine/threonine-specific protein kinase, serves as the central downstream effector of PI3K signaling. It comprises 3 isoforms: AKT1 (PKBα), AKT2 (PKBβ), and AKT3 (PKBγ).^[166]^

Full activation of AKT requires phosphorylation at 2 key sites: Thr308 within the kinase activation loop and Ser473 in the C-terminal regulatory domain.^[166]^

### 3.2. Canonical activation cascade

**Figure 2.**
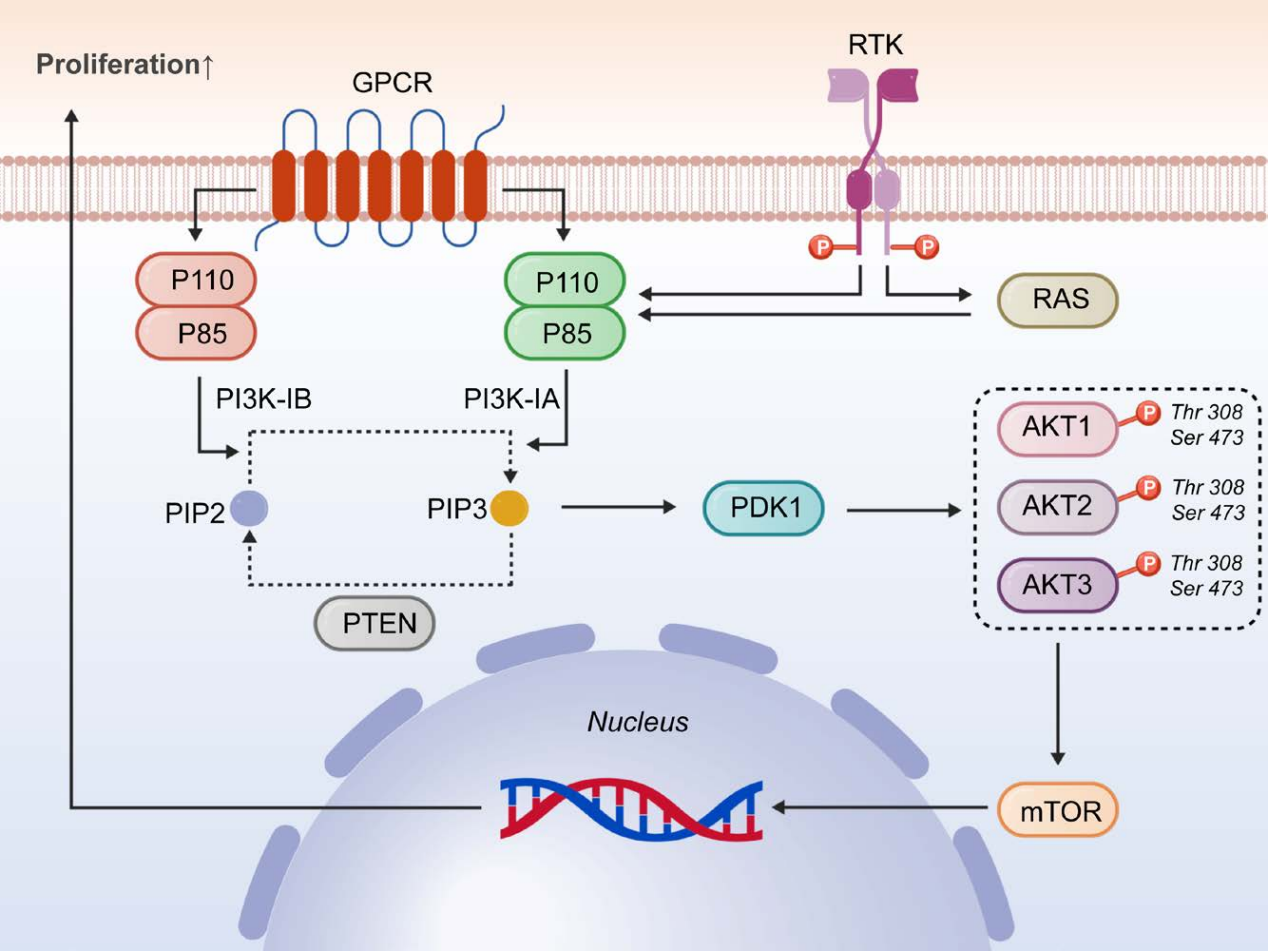
Structural composition and activation of the PI3K/AKT pathway. PI3K = phosphatidylinositol 3-kinase.

The canonical activation of the PI3K/AKT pathway is initiated at the plasma membrane. Following ligand binding (e.g., to VEGFR), RTKs undergo autophosphorylation, creating docking sites for the regulatory subunit of PI3K. This recruitment relieves the autoinhibition on the PI3K catalytic subunit, enabling it to phosphorylate the membrane lipid phosphatidylinositol 4,5-bisphosphate to generate phosphatidylinositol 3,4,5-trisphosphate.^[167]^ Phosphatidylinositol 3,4,5-trisphosphate acts as a critical second messenger, recruiting pleckstrin homology domain-containing proteins, including AKT and its upstream activator PDK1, to the membrane. PDK1 phosphorylates AKT at Thr308, while mTOR complex 2 typically mediates phosphorylation at Ser473, leading to full AKT activation.^[168]^ Subsequently, activated AKT phosphorylates a multitude of downstream targets, such as the mTOR, to regulate essential cellular processes including growth, proliferation, metabolism, and survival^.[169]^ (Fig. 2).

## 4. The integrative role of the PI3K/AKT signaling pathway as a “dynamic command hub” in NSCLC

The PI3K/AKT signaling pathway in NSCLC functions not merely as a simple linear signal transducer but rather as a core “dynamic command hub.” It integrates diverse upstream signals and temporally coordinates downstream functional modules across different stages of tumor progression to drive malignant evolution.

## 5. The PI3K/AKT signaling pathway as a dynamic command hub: integrative functions in NSCLC

This pathway serves as a convergence point for multiple oncogenic signals. Beyond classical activation by growth factors such as EGFR,^[170]^ it is regulated by diverse factors within the TME, including oxidative stress (e.g., NOX4-derived ROS activating the pathway^[171]^), chronic inflammation (e.g., FGFR1/Toll-like receptor 4 co-driving cytokine storms^[172]^), and stromal cell interactions (e.g., senescent fibroblasts activating the pathway via MMP1 secretion^[173]^). Collectively, these signals form a persistent activation network, and the pathway itself undergoes self-amplification through positive feedback loops – such as promoting EGFR transcription via NF-κB^[174]^ or relieving EGFR negative regulation by destabilizing phosphatase and tensin homolog (PTEN)^[175]^ – thereby establishing a foundation for sustained activation.

### 5.1. Temporal regulation of malignant phenotypes by the PI3K/AKT functional command hub

Activated PI3K/AKT signaling orchestrates the entire process of tumor progression through the precise regulation of downstream substrates:

Initiating proliferation and survival: In early tumorigenesis, the pathway establishes a growth advantage by driving the cell cycle (promoting G1/S transition^[176]^) and inhibiting apoptosis (modulating the Bcl-2/Bax ratio^[177,178]^). Concurrently, it provides multiple safeguards for cell survival by suppressing autophagy^[177]^ and resisting pyroptosis.^[178]^

Promoting adaptation and invasion: As the tumor grows, the pathway induces EMT and upregulates MMPs to enhance cell migration and invasion, enabling the tumor to overcome physical constraints.^[179]^ Furthermore, it promotes angiogenesis via mechanisms such as HIF-1α activation to supply nutrients for expansion.

Shaping an immunosuppressive microenvironment: In advanced stages, the pathway shifts its focus to immune editing, recruiting immunosuppressive cells via factors like interleukin-8 and inhibiting effector T cell function, thereby creating a permissive environment for immune escape and distant metastasis.^[180]^

### 5.2. Formation of a self-sustaining malignant cycle: locking in tumor progression

Critically, the downstream processes driven by the PI3K/AKT pathway – such as oxidative stress, inflammation, and hypoxia – themselves serve as potent stimuli that reactivate the pathway.^[171]^ This multi-layered positive feedback loop (e.g., the inflammation-pathway activation cycle, the hypoxia–angiogenesis cycle) forms a self-sustaining “malignant engine” that not only locks in the malignant phenotype but also drives continuous progression to more advanced stages.^[181]^

### 5.3. Clinical evidence and therapeutic implications

The central role of this pathway is clinically validated, with frequent aberrant activation observed in NSCLC, such as *PIK3CA* mutations^[182]^ and elevated Akt activity.^[183,184]^ This makes it a highly promising therapeutic target. Studies have confirmed that targeting this pathway not only inhibits proliferation but also triggers a unique form of caspase-3/gasdermin E-dependent pyroptosis,^[178]^ offering a novel approach to overcoming resistance to conventional therapies. Understanding its integrative function as a “dynamic command hub” is crucial for developing precision combination strategies that simultaneously block its upstream activation, downstream effectors, and feedback loops.

**Figure 3.**
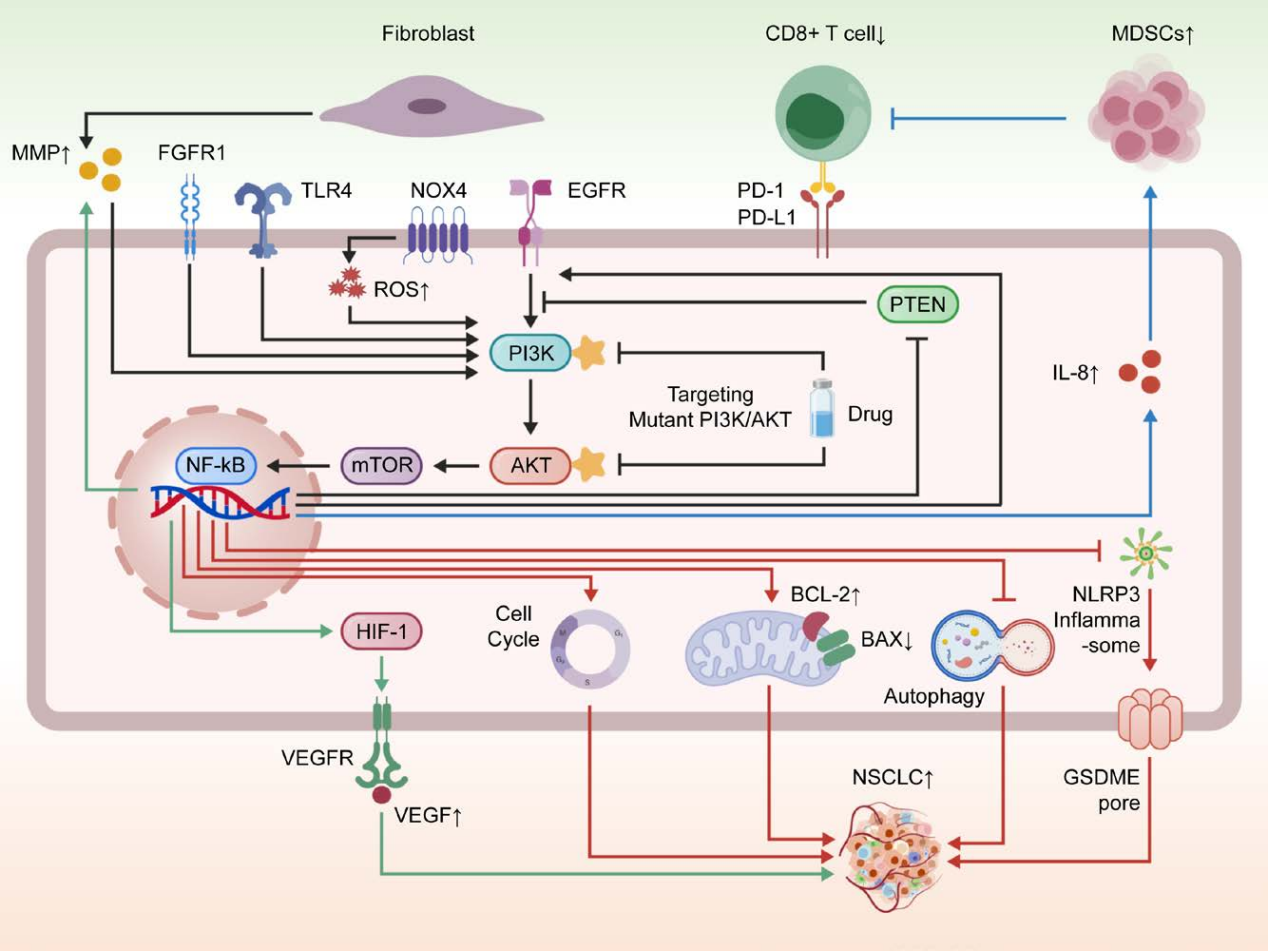
The integrative role of the PI3K/AKT signaling pathway as a “dynamic command hub” in NSCLC. NSCLC = non-small cell lung cancer; PI3K = phosphatidylinositol 3-kinase.

Consequently, targeting the PI3K/AKT signaling pathway has emerged as a critical therapeutic strategy for NSCLC, given its central role in regulating cell proliferation, survival, and metabolism. By inhibiting aberrant pathway activation, it is possible to effectively block tumor growth and enhance chemosensitivity, offering new therapeutic hope for NSCLC patients (Fig. 3).

#### 5.3.1. Modulation of NSCLC progression by active herbal monomers

Despite significant advances in NSCLC treatment modalities – including targeted therapy, chemotherapy, immunotherapy, and surgical resection – clinical outcomes remain limited. While surgical resection remains the primary treatment for early-stage NSCLC, postoperative inflammatory responses may promote TME remodeling and increase circulating tumor cell dissemination, thereby elevating hematogenous metastasis risk.^[185]^ Moreover, high postoperative recurrence rates (particularly in locally advanced cases) pose retreatment challenges that substantially compromise survival outcomes.^[186]^ TCM, with millennia of clinical application in Asia, demonstrates unique advantages in cancer adjuvant therapy through its holistic regulation and syndrome differentiation-based treatment principles. In NSCLC treatment, TCM exerts multi-component and multi-target effects that effectively alleviate chemoradiotherapy toxicity, enhance immune function, suppress TME inflammation, and potentially reduce postoperative recurrence risk.^[187]^ TCM demonstrates potential therapeutic value in NSCLC treatment, though its multi-component and multi-target nature presents challenges in fully elucidating the underlying mechanisms. Therefore, elucidating the molecular mechanisms by which active herbal components modulate NSCLC through the PI3K/AKT signaling pathway is of significant importance. Based on the specific mechanisms by which herbal monomers inhibit tumor growth, metastasis, and drug resistance through intervention in the PI3K/AKT pathway, this review aims to provide a theoretical basis and research framework for developing novel targeted anti-NSCLC drugs.

## 6. Compounds directly targeting the core components of the PI3K/AKT pathway

### 6.1. Curcumol

Evidence chain: Curcumol exhibits anti-NSCLC activity in vitro (A549, H460 cells) and in vivo (A549 xenograft models), inhibiting proliferation, migration, and invasion. Mechanistically, it dose-dependently downregulates phosphorylated PI3K and AKT levels, directly targeting the core nodes of the pathway, while concurrently suppressing Wnt/β-catenin signaling. These actions induce G0/G1 cell cycle arrest, mitochondrial apoptosis, and downregulation of MMPs.^[186]^

Synergistic potential: Curcumol demonstrates significant synergy with the COX-2 inhibitor celecoxib. The combination more effectively suppresses the PI3K/AKT and ERK/mitogen-activated protein kinase (MAPK) pathways, inhibits NF-κB nuclear translocation, and modulates apoptosis regulators (Bcl-2/Bax), leading to enhanced antitumor effects in vitro and in vivo without increased toxicity.^[188]^

Critical translational gaps: The effective in vitro concentrations for curcumol alone (~80 µM) and in combination (30 µM) are relatively high. There is a complete lack of pharmacokinetic data (e.g., oral bioavailability, half-life) for curcumol monotherapy and no drug–drug interaction studies for the combination, preventing correlation between in vitro potency and achievable in vivo exposure. Its direct molecular target within the PI3K/AKT pathway remains uncharacterized. Compared to classical PI3K/AKT inhibitors, curcumol’s multi-target profile may increase the risk of off-target effects.^[186,188]^

### 6.2. Fucoxanthin (FX)

Evidence chain: Fucoxanthin inhibits proliferation, migration, and invasion of NSCLC cells (NCI-H1299, A549) in vitro and suppresses tumor growth in A549 xenograft models in vivo. Rescue experiments using PI3K/AKT agonists (IGF-1, SC-79) confirm that its effects are mediated through direct inhibition of PI3K and AKT phosphorylation.^[189]^

Synergistic potential: FX enhances the sensitivity of NSCLC to gefitinib both in vitro and in vivo, associated with upregulation of tissue inhibitor of metalloproteinases-2. The combination shows superior efficacy without increased systemic toxicity.^[190]^

Critical translational gaps: The effective in vitro concentrations (half maximal inhibitory concentration [IC_50_]; 17–41 µM; 15 µM in combination) are high. No pharmacokinetic data are available to bridge in vitro and in vivo doses. The selective toxicity of FX toward normal cells has not been assessed, raising concerns about potential on-target toxicity in healthy tissues. Pharmacokinetic interaction data for the FX-gefitinib combination are also lacking.^[189,190]^

### 6.3. Quercetin

Evidence chain: Quercetin exerts anti-NSCLC effects via distinct, well-validated mechanisms. One study demonstrates it directly binds to and upregulates SIRT5, which inhibits PI3K phosphorylation and downstream AKT activation, leading to DNA damage accumulation and apoptosis.^[191]^ Separately, quercetin overcomes EGFR T790M-mediated resistance by competitively inhibiting glucose-6-phosphate dehydrogenase, inducing oxidative degradation of the mutant EGFR protein. It also synergizes with gefitinib in EGFR T790M-mutant models.^[192]^ Notably, quercetin exhibited no significant inhibitory effect on human normal lung epithelial cells (BEAS-2B) at its effective concentrations, suggesting a favorable selectivity towards NSCLC cells.

Critical translational gaps: Its effective concentration in the SIRT5/PI3K study is high (12.5–200 µM), while it shows lower µM potency in the glucose-6-phosphate dehydrogenase/EGFR study. Systematic pharmacokinetic data are lacking, despite reported in vivo efficacy. The relationship between its multiple mechanisms and primary anticancer effect in NSCLC requires clarification.^[191,192]^

### 6.4. Hydroxysafflor Yellow A (HSYA)

#### Evidence chain

HSYA inhibits lipopolysaccharide-induced proliferation, migration, invasion, and EMT in human NSCLC cells (A549 and H1299). The underlying mechanism is associated with the simultaneous and direct suppression of the PI3K/Akt/mTOR and ERK/MAPK signaling pathways. This conclusion was verified using specific pathway inhibitors (LY294002 and SCH772984). Co-treatment with HSYA and either inhibitor exerted more potent anticancer effects than either agent alone.^[193]^

#### Critical translational gaps

HSYA demonstrates promising NSCLC activity in vitro; however, all current evidence is based exclusively on cellular models, with no in vivo pharmacodynamic validation. The effective concentrations observed in vitro (5–20 µM) are relatively high, and no pharmacokinetic parameters have been reported, making it difficult to predict whether therapeutically relevant concentrations can be achieved and sustained in vivo. Furthermore, the inhibitory effects of HSYA on the PI3K/Akt/mTOR and ERK/MAPK signaling pathways are downstream events, and its direct molecular target(s) remain unidentified. This lack of defined target(s) hampers structure-based optimization for enhanced selectivity and limits the evaluation of potential off-target toxicity.^[193]^

### 6.5. Dieckol

Evidence chain: dieckol inhibits the proliferation, migration, and invasion of NSCLC A549 cells in vitro, with a median lethal concentration (LC_50_) of approximately 25 µg/mL. Its antitumor mechanism involves the direct suppression of the PI3K/AKT/mTOR signaling pathway and the reversal of EMT. Specifically, dieckol dose-dependently downregulates the protein levels of phosphorylated PI3K, phosphorylated AKT, and phosphorylated mTOR, while upregulating the expression of the tumor suppressor protein E-cadherin. Concurrently, it downregulates the expression of pro-metastatic and pro-invasive proteins, including N-cadherin, vimentin, TWIST, and MMP-9.

Critical translational gaps: Current evidence supporting the antitumor activity of dieckol is exclusively obtained from in vitro experiments using A549 NSCLC cells, with no in vivo studies to validate its efficacy, safety, or tissue distribution. The in vitro effective concentration (LC_50_ = 25 μg/mL) is relatively high, and no pharmacokinetic parameters – including oral bioavailability, plasma half-life, tissue accumulation, or metabolic profile – have been reported. These gaps greatly limit the evaluation of its translational potential for in vivo application.^[194]^.

The monomers discussed here, demonstrate that directly targeting the PI3K/AKT pathway can yield compelling preclinical efficacy, including promising combination synergyn (Table 1).

**Table 1:** Summary of herbal monomers directly targeting the PI3K/AKT pathway core components.

Compound (class)	Key model (s)	Proposed direct mechanism	In vitro efficacy	In vivo efficacy	Major translational gaps
Curcumol^[186]^	A549, H460 cells; nude mouse xenograft model	Inhibits the phosphorylation of PI3K (p‑PI3K) and AKT (p‑AKT); suppresses the phosphorylation of the Wnt co‑receptor LRP5/6, enhances β‑catenin phosphorylation to promote its degradation, and thereby inhibits the activation of both signaling pathways.	20–80 µmol/L	100, 200, and 300 mg/kg	High in vitro conc.; no PK data; target unknown; no DDI data for combo.
Fucoxanthin^[189]^	A549 and NCI-H1299 cells; nude mouse xenograft tumor model	Inhibits PI3K/Akt phosphorylation (rescued by IGF-1/SC-79)	25–50 µM IC_50_ ≈ 25 µg/mL	37.5, 70, and 150 mg/kg	High in vitro conc.; no PK data; selectivity unassessed; no DDI data for combo.
Quercetin	BEAS-2B, A549, and H1299 cells; LLC xenograft tumor	1. Activates SIRT5 and thereby inhibits the phosphorylation of PI3K and AKT. 2. Quercetin promotes the degradation of the EGFR T790M mutant by competitively inhibiting the binding of G6PD with NADP^+^.	12.5–200 µM^[191]^; 1–20 µM; IC_50_: 15–25 µM^[192]^	200 mg/kg^3^; 40 mg/kg^4^	High conc. for Mech 1; lack of systematic PK data; multi-mechanism interplay unclear.
Hydroxysafflor Yellow A^[193]^	A549, H1299 cells	Inhibits the phosphorylation of PI3K, AKT, ERK, and MAPK.	5–20 μM	NA	In vitro only, high effective concentration, no pharmacokinetic data.
Dieckol^[194]^	A549 cell	Inhibits p-PI3K, p-Akt, p-mTOR; reverses EMT	25–50 μM IC_50_ ≈ 25 µg/mL	NA	In vitro only, high concentration, no pharmacokinetic data

Combo. *=* combination, conc. *=* concentration, DDI *=* drug–drug interaction, PK *=* pharmacokinetics.

## 7. Compounds indirectly affecting the PI3K/AKT pathway via modulation of upstream receptors (e.g., EGFR, c-Met)

### 7.1. Dictamnine

Evidence chain: dictamnine (Dic) exerts anti-NSCLC effects by binding to the ATP-binding site of the c-Met receptor via hydrogen bonds, inhibiting its kinase activity. This inhibition dually suppresses the downstream PI3K/Akt/mTOR and MAPK pathways. Dic effectively inhibits proliferation, migration, invasion, and induces apoptosis in vitro (A549, H1299, PC9GR cells) and in vivo (A549 xenografts).

Synergistic potential: notably, Dic synergistically enhances the efficacy of EGFR tyrosine kinase inhibitors gefitinib and osimertinib against EGFR-tyrosine kinase inhibitor-resistant PC9GR cells (combination index < 1), offering a novel strategy to overcome acquired resistance.

Critical translational gaps: Its effective concentration in vitro covers a broad and relatively high range (IC_50_ ≈ 2.8–200 μM). There is a complete absence of pharmacokinetic data despite demonstrated in vivo efficacy at 50 mg/kg. Compared to selective c-Met inhibitors (e.g., capmatinib), Dic’s potential multi-target nature may result in lower potency and specificity. Comprehensive absorption, distribution, metabolism, excretion (ADME) and safety studies, as well as validation in more clinically relevant models (e.g., patient-derived xenografts), are required.^[195]^

### 7.2. Shikonin

Evidence chain: shikonin suppresses migration and invasion in NSCLC cells by inhibiting c-Met phosphorylation and its downstream PI3K/Akt and MEK/ERK signaling, thereby reversing EMT. It effectively inhibits both endogenous c-Met activation in HCC827 cells and HGF-induced c-Met activation in A549 cells, providing a coherent mechanistic narrative.

Critical translational gaps: A key limitation is the absence of direct target validation (e.g., via CETSA, DARTS). The mechanism remains correlative, unable to definitively distinguish between direct c-Met inhibition and indirect modulation. Furthermore, the study lacks in vivo efficacy data, severely limiting translational assessment. Although its effective concentration in vitro is relatively low (0.25–1 µM) and it shows specific anti-migratory activity at non-cytotoxic doses, its full therapeutic potential remains uncharacterized without in vivo validation.^[196]^

This section reviews herbal monomers that target upstream RTKs, such as c-Met, leading to the indirect suppression of the downstream PI3K/AKT pathway, and evaluates their potential in overcoming targeted therapy resistance(Table 2).

**Table 2:** Summary of herbal monomers targeting the PI3K/AKT pathway via upstream receptors.

Compound (class)	Proposed primary target/mechanism	Key model (s)	In vitro efficacy	In vivo efficacy	Major translational gaps
Dictamnine^[195]^	Binds to c-Met ATP-binding site; inhibits PI3K/Akt and MAPK expression.	A549, NCI‑H1299, PC9GR, EBC‑1 cells; A549 xenograft model in nude mice	50–200 μM Synergistic effect: 12.5–200 μM IC_50_ ≈ 2.811 μM	50 mg/kg	High in vitro effective concentration; no pharmacokinetic data
Shikonin^[196]^	Inhibits c-Met phosphorylation; suppresses PI3K/Akt and MEK/ERK transcription	H23, A549, and HCC827 cell	0.25–1 μM inhibits c-Met phosphorylation; 2 μM induces apoptosis	NA	Lack of direct target evidence; no in vivo efficacy; unclear mechanistic correlation

Conc. *=* concentration, PK *=* pharmacokinetics.

## 8. Compounds modulating the PI3K/AKT pathway via microRNA networks

### 8.1. Anwulignan (ANW)

Evidence chain: ANW enhances the efficacy of cisplatin by upregulating let-7c-3p, a miRNA that directly targets the 3′-UTR of *PIK3CA* (encoding the p110α subunit of PI3K). This leads to suppression of the PI3K/AKT/mTOR pathway, resulting in G0/G1 cell cycle arrest and, in combination with cisplatin, synergistic induction of apoptosis and reversal of EMT in vitro. Co-administration of ANW with cisplatin significantly inhibits tumor growth in xenograft models without evident organ toxicity.

Critical translational gaps: A major mechanistic “black box” exists: the upstream mechanism by which ANW upregulates let-7c-3p is entirely unknown. Furthermore, despite in vivo efficacy, essential pharmacokinetic data (e.g., bioavailability, half-life) are completely lacking, preventing any assessment of its druggability. The identification of ANW’s direct molecular target is required to elucidate the initial step of its action.^[197]^

### 8.2. Icariin (ICA)

Evidence chain: ICA suppresses lung cancer progression by downregulating the oncomiR miR-205-5p, which directly targets the 3′-UTR of the tumor suppressor PTEN. By relieving this repression, ICA elevates PTEN levels, leading to inhibition of the PI3K/Akt pathway. It inhibits proliferation, migration, invasion, and induces apoptosis in vitro (A549, H1975 cells) with minimal cytotoxicity to normal bronchial epithelial cells (BEAS-2B). In vivo, ICA (10 mg/kg) significantly suppresses tumor growth and proliferation in a xenograft model.

Critical translational gaps: The effective concentration in vitro (20 µM) is relatively high, and there is a complete lack of pharmacokinetic data, making it impossible to correlate the in vitro activity with achievable human plasma levels. Additionally, while the miR-205-5p/PTEN axis is established, its potential downstream immunomodulatory consequences (e.g., on PD-L1 expression) remain unexplored, leaving a gap in understanding its full impact on the tumor immune microenvironment.^[198]^

### 8.3. Sulforaphene (SFE)

Evidence chain: SFE inhibits the PI3K/AKT pathway by downregulating multiple oncogenic microRNAs (miRNAs) (miR-10a, –205, −221, −222) that target *PTEN*, thereby increasing PTEN protein levels. It shows broad in vitro efficacy across 6 NSCLC cell lines and inhibits tumor growth in vivo (A549 xenograft).

Notable translational strength: This study provides a rare and complete preclinical package for a natural product, including systematic acute toxicity (LD_50_) and comprehensive pharmacokinetic data (oral bioavailability, maximum plasma concentration, *T*_max_, half-life), offering strong preliminary evidence of druggability.

Critical translational gap: Despite the advanced pharmacological profiling, the direct molecular target of SFE remains unidentified. The upstream mechanism controlling the panel of miRNAs is a “black box” similar to ANW. Its indirect, multi-miRNA action may reduce specificity compared to direct kinase inhibitors.^[199]^

This section reviews herbal monomers that exert their anti-NSCLC effects by modulating specific miRNAs, which in turn regulate key components of the PI3K/AKT pathway, offering a unique layer of epigenetic intervention (Table 3).

**Table 3:** Summary of herbal monsters modulating the PI3K/AKT pathway via miRNA networks.

Compound (class)	Key miRNA(s) regulated	Downstream effect on PI3K/AKT	Key model(s)	In vitro efficacy	In vivo efficacy & PK/Tox	Major translational gaps
Anwulignan^[197]^	Upregulates the expression of let-7c-3p	Targets PIK3CA 3′-UTR; inhibits the signaling pathway	A549 and H460 cells; A549 xenograft tumor model in nude mice	20–50 μM	20 mg/kg	High in vitro concentration; unknown upstream mechanism; no PK data
Icariin^[198]^	Inhibits miR-205-5p	Relieves PTEN-mediated inhibition; thereby suppressing the pathway	A549, NCI-H1975 and BEAS-2B cells; nude mouse xenograft tumor model	5–40 μM	10 mg/kg	High in vitro concentration; no pharmacokinetic data; unknown immunomodulatory effect
Sulforaphene^[199]^	Inhibits miR-10a, −205, −221, −222	Relieves PTEN-mediated inhibition; thereby suppressing the pathway	Six NSCLC cell lines; A549 xenograft	10–40 μM	Intravenous administration: 50 mg/kg; oral administration: 100 mg/kg. Acute toxicity: LD_50_ = 202.7 mg/kg	Unknown target

Combo. *=* combination, conc. *=* concentration, PK *=* pharmacokinetics, Tox *=* toxicology.

## 9. Compounds inhibiting the PI3K/AKT pathway via induction of oxidative stress

### 9.1. Plumbagin (PLB)

Evidence chain: PLB exerts anti-NSCLC effects by inducing a massive generation of intracellular ROS, which triggers oxidative stress and subsequently inhibits the PI3K/Akt/mTOR signaling pathway. This leads to the concurrent activation of caspase-dependent mitochondrial apoptosis and autophagic cell death in both p53-wild-type (A549) and p53-mutant (H23) cells. The ROS burst causes G2/M phase arrest and disrupts mitochondrial integrity, activating the caspase cascade. The central role of ROS is confirmed by reversal with the antioxidant N-acetylcysteine. PLB also shows synergistic enhancement of autophagy when combined with the vascular disrupting agent DMXAA.

Critical translational gaps: The mechanism remains at the level of phenotypic correlation, lacking direct target validation. Its effective concentration in vitro is relatively high (IC_50_: 7–10 μM). Most critically, the study completely lacks in vivo efficacy and pharmacokinetic data, casting serious doubt on its clinical feasibility.^[200]^

### 9.2. Juglone

Evidence chain: Juglone induces a substantial intracellular increase in ROS, which in turn inhibits the PI3K/Akt signaling pathway. It induces apoptosis and suppresses proliferation, migration, and invasion in vitro and in a Lewis lung carcinoma allograft model. Gain- and loss-of-function experiments using a PI3K/Akt agonist and the antioxidant N-acetylcysteine established the causal “ROS → PI3K/Akt → apoptosis” sequence.

Critical translational gaps: A significant paradox exists: low in vitro potency (IC_50_: 10 μM) contrasts with a relatively high effective in vivo dose (10 mg/kg), strongly suggesting extremely poor pharmacokinetic properties (e.g., low oral bioavailability, rapid clearance), yet no pharmacokinetics data are available. The direct molecular target for ROS induction is unknown, making it a nonspecific oxidative stress inducer with potential off-target risks compared to selective inhibitors.^[201]^

### 9.3. Resveratrol

Evidence chain: Resveratrol demonstrates context-dependent mechanisms. As a single agent in SCLC cells, it induces ROS to inhibit the PI3K/Akt/c-Myc axis and apoptosis.^[202]^ In combination with cisplatin, it enhances apoptosis by activating autophagy and inhibiting PI3K/AKT.^[203]^ With arsenic trioxide, it synergistically elevates ROS to activate multiple apoptotic pathways.^[204]^

Critical translational gaps: All evidence for resveratrol is confined to in vitro cell models (A549, H446), with a complete absence of in vivo efficacy data in NSCLC. The effective concentrations range widely (2.5–80 µM) and are relatively high, with no supporting pharmacokinetic data to assess achievable plasma levels.^[202–204]^

### 9.4. Berberine (BER)

Evidence chain: BER activity is prominent in combinatorial settings. It synergizes with cold atmospheric plasma in vitro, cooperatively downregulating PI3K/AKT and elevating ROS.^[205]^ Separately, BER synergizes with sulfasalazine (SAS) in vitro and in vivo (LLC-1 allograft) to induce ferroptosis via the p53/SLC7A11/GPX4 axis, involving ROS accumulation and GSH depletion.^[206]^

Critical translational gaps: The effective in vitro concentration for BER (20 µM) is high, and pharmacokinetic data are lacking for both combination strategies. The innovative BER-cold atmospheric plasma approach lacks in vivo efficacy and safety data, and its tumor selectivity is unknown. The promising BER–SAS combination still requires detailed drug–drug interaction and safety studies.^[205,206]^

This section reviews herbal monomers that exert anti-NSCLC effects primarily by inducing a burst of intracellular ROS, which subsequently leads to the inhibition of the PI3K/AKT pathway, triggering various forms of cell death (Table 4).

**Table 4:** Summary of herbal monomers inhibiting the PI3K/AKT pathway via ROS induction.

Compound (class)	Key mechanism/ context	Key model(s)	In vitro efficacy	In vivo efficacy	Major translational gaps
Plumbagin^[200]^	Induces ROS burst, thereby inhibiting PI3K/Akt/mTOR pathway and triggering tumor cell apoptosis and autophagy。	A549, H23 cells	Concentration range: 0.5–10 μM IC_50_ (A549): 10.87 μM IC_50_ (H23): 7.80 μM	NA	No in vivo data; no pharmacokinetic data
Juglone^[201]^	Induces ROS burst, inhibits PI3K/Akt, and activates mitochondrial apoptosis	A549 and LLC cells; A549 xenograft tumor model in nude mice	Concentration range: 2–8 μM IC_50_ (LLC cells): 10.78 μM IC_50_ (A549 cells): 9.47 μM	10 mg/kg	No pharmacokinetic data
Resveratrol	Activates ROS and inhibits the PI3K/Akt signaling pathway	A549, H446 cells , A549 xenograft tumor model in nude mice	40 μg/mL^[202]^; Monomer: 2.5–40 μM; IC_50_ = 8.3 μM; combination with cisplatin: resveratrol 2.5 and 60 μM^[203]^; 60 μM^[204]^	No in vivo concentration data	No pharmacokinetic data; high concentration with large variation
Berberine	Combination: synergizes with LTP/SAS, inhibits PI3K/AKT, increases ROS/ferroptosis	A54 and H1299cells; SAS combination: LLC-1 allograft	20 µM^[205]^; SAS combination:5–75 µM^[206]^	5mg/kg^18^	High concentration in vitro; no pharmacokinetic data.

Combo. *=* combination, conc *=* concentration, LTP *=* cold atmospheric plasma, PK *=* pharmacokinetics, SAS *=* sulfasalazine.

## 10. Compounds suppressing tumors via coordinated modulation of mitochondrial function and EMT pathways

### 10.1. Vitexin

Evidence chain: Vitexin induces mitochondrial dysfunction and inhibits the PI3K/Akt/mTOR signaling pathway, effectively promoting apoptosis in NSCLC cells in vitro and in an A549 xenograft model. Mechanistically, it decreases the Bcl-2/Bax ratio, activates caspase-3, and triggers mitochondrial membrane potential collapse and cytochrome c release. The role of the PI3K/Akt/mTOR axis is supported by partial rescue with an AKT agonist (SC-79).

Notable translational advantage: Vitexin exhibits selective cytotoxicity, effectively inhibiting A549 cell viability (IC_50_: 10–40 μM) while showing minimal toxicity toward normal human bronchial epithelial cells (16HBE).

Critical translational gaps: Its effective concentration in vitro is relatively high (≥10 μM). There is a complete lack of pharmacokinetic data, preventing correlation between the in vitro potency and the in vivo effective dose (1–2 mg/kg) and raising concerns about druggability. The direct molecular target responsible for PI3K/Akt/mTOR inhibition remains unidentified.^[207]^

### 10.2. Cardamonin

Evidence chain: Cardamonin directly inhibits the PI3K/Akt/mTOR pathway and, critically, reverses EMT by upregulating E-cadherin and downregulating N-cadherin and the transcription factor Zinc Finger E-Box Binding Homeobox 1. It suppresses proliferation, migration, invasion, induces G2/M arrest and apoptosis in vitro across 5 NSCLC cell lines, and inhibits tumor growth in an H460 xenograft model, presenting a complete evidence chain from molecular inhibition to in vivo efficacy.

Notable translational advantage: Cardamonin offers a unique anti-metastatic profile via EMT reversal, a mechanism distinct from classical PI3K/Akt inhibitors that primarily target proliferation and survival.

Critical translational gaps: Similar to vitexin, its in vitro IC_50_ is relatively high (≈10–20 μM), and it lacks systematic pharmacokinetic data (e.g., bioavailability, half-life), hindering dose translation. Its potency and specificity may be lower compared to highly selective inhibitors.^[208]^

This section reviews herbal monomers that exert anti-NSCLC effects by concurrently targeting the PI3K/AKT/mTOR pathway and distinct secondary processes – specifically, mitochondrial apoptosis and EMT – representing a dual-pathway intervention strategy (Table 5).

**Table 5:** Summary of herbal monomers targeting PI3K/AKT with coordinated secondary mechanisms.

Compound (class)	Primary action (PI3K/AKT)	Key secondary mechanism	Key model(s)	In vitro efficacy	In vivo efficacyy	Major translational gaps
Vitexin^[207]^	Inhibition of the PI3K/Akt/mTOR signaling pathway	Induction of mitochondrial apoptosis	A549, 16 HBE cells and A549 xenografts	10–40 µM	1–2 mg/kg	High concentration in vitro; no pharmacokinetic data available.
Cardamonin^[208]^	Inhibition of the PI3K/Akt/mTOR signaling pathway	Inhibits proliferation and induces apoptosis; inhibits migration and invasion	A549 and H460 cells; H460 xenografts	0–40 µM	5 mg/kg	High concentration in vitro; no pharmacokinetic data available.

Conc = concentration, PK = pharmacokinetics.

## 11. Clinical perspective: validated PI3K/AKT pathway inhibitors in NSCLC therapy – a benchmark for herbal monomer development

### 11.1. Paclitaxel: a clinically validated agent with PI3K/AKT-modulating activity

Paclitaxel, a cornerstone microtubule-targeting chemotherapeutic, exemplifies a clinically validated drug whose multifaceted mechanism of action includes the modulation of the PI3K/AKT pathway. Clinically, as part of a neoadjuvant chemoimmunotherapy regimen with carboplatin and nivolumab, it has demonstrated remarkable efficacy in resectable stage IIIA NSCLC. The NADIM study reported a major pathological response rate of 83%, a pathological complete response rate of 63%, and impressive 24-month progression-free survival and OS rates of 77.1% and 89.9%, respectively, following this combination.^[209]^

Mechanistically, beyond its primary action of stabilizing microtubules, paclitaxel induces a ROS burst leading to DNA damage and directly inhibits the EGFR/PI3K/AKT/mTOR survival signaling axis. This contributes to the inhibition of proliferation, G1 cell cycle arrest, and mitochondrial apoptosis in EGFR-mutant NSCLC cells in vitro. The pivotal role of ROS is supported by reversal experiments with the antioxidant N-acetylcysteine, and the study further delineated its impact on the DNA damage response (ATR/Chk1, γ-H2AX) and the concomitant suppression of pro-survival pathways.^[210]^

### 11.2. Implications for herbal monomer development

The case of paclitaxel serves as a critical benchmark for evaluating the developmental potential of PI3K/AKT-modulating herbal monomers discussed in this review. It underscores several key principles:

#### 11.2.1. The primacy of clinical evidence

Compelling preclinical mechanistic data (including PI3K/AKT pathway inhibition) is a necessary but insufficient foundation. Robust clinical trial evidence is the ultimate determinant of therapeutic success.

#### 11.2.2. The value of multi-mechanistic action

Profound therapeutic efficacy can arise from a multi-targeted pharmacodynamic profile. Paclitaxel’s efficacy derives from its primary action on microtubules, combined with secondary effects on key survival pathways (e.g., PI3K/AKT) and oxidative stress. This mirrors the multi-pathway modulation observed with many herbal monomers (e.g., curcumol, quercetin), suggesting their complex pharmacology could be a potential asset rather than a liability, if properly harnessed.

#### 11.2.3. The translational path defined by pharmacokinetics and safety

The clinical utility of paclitaxel is underpinned by well-characterized pharmacokinetics, a defined safety profile, and optimized administration regimens. This highlights the most significant gap for the herbal monomers: the near-universal lack of pharmacokinetic data and systematic safety assessment, which are prerequisites for advancing any compound into clinical trials.

The case of paclitaxel establishes a critical developmental paradigm for the herbal monomers reviewed herein. First, it underscores the primacy of clinical evidence; compelling preclinical mechanism, while necessary, is ultimately validated and superseded by robust outcomes from rigorous clinical trials. Second, it highlights the therapeutic value of a multi-targeted pharmacodynamic profile. Paclitaxel’s efficacy arises from the synergy between its primary mechanism and secondary effects on pathways like PI3K/AKT – a property that mirrors the complex, multi-pathway pharmacology of many herbal monomers (e.g., curcumol, quercetin), suggesting their inherent polypharmacology could be a strategic asset if rationally developed. Most critically, it defines the non-negotiable translational pathway built on pharmacokinetics and safety. Paclitaxel’s clinical utility is predicated on well-characterized ADME properties and a managed safety profile, which starkly contrasts with the most significant near-universal gap for the discussed herbal monomers: the profound lack of pharmacokinetic data and systematic toxicological assessment.

Therefore, paclitaxel serves not only as a benchmark for success but also as a clear roadmap. For herbal monomers to transition from promising preclinical entities to viable therapeutics, future efforts must rigorously emulate this pathway: advancing through comprehensive ADME/Tox profiling to de-risk clinical translation, and strategically pursuing rational combination therapies – as exemplified by paclitaxel’s own evolution within chemoimmunotherapy regimens – to maximize their therapeutic potential within the complex landscape of NSCLC treatment.

## 12. Discussion

The pathogenesis of NSCLC is highly complex, involving dynamic interactions among genetic mutations, epigenetic regulation, inflammatory microenvironments, and metabolic reprogramming. In this context, TCM monomers, characterized by their well-defined structures and analyzable targets, have emerged as a promising strategy to overcome the limitations of conventional therapies. Existing research has confirmed that monomeric components such as baicalein and triptolide can specifically inhibit key nodes of the PI3K/AKT pathway, significantly suppressing tumor proliferation and reversing drug resistance, thereby providing lead compounds for precision therapy.

However, 2 critical gaps remain in current research: insufficient exploration of the gut–lung axis mechanism.

Recent studies have found that the gut microbiota can remotely regulate the pulmonary immune microenvironment and inflammatory status through metabolites and secretions, with the PI3K/AKT signaling pathway potentially serving as a core mediator in microbiota–host interactions. For instance, research has explored how cryptotanshinone alleviates pulmonary inflammation and fibrosis in a mouse model by modulating gut microbiota dysbiosis and bile acid metabolism from the perspective of the gut–lung axis.^[211]^ However, such studies have not directly demonstrated its relevance in regulating lung cancer development via the PI3K/AKT pathway. Future investigations need to integrate metagenomics and metabolomics to systematically decipher the regulatory network of “TCM monomers–gut microbiota–lung cancer” to fill this knowledge gap.

Urgent need for deepened structure–activity relationship (SAR) studies. Although the mechanisms of action for some monomers have been elucidated, most components remain at the stage of phenotypic observation, with their direct molecular targets and efficient SAR still unclear. Therefore, the following strategies are recommended for breakthrough:

1. Computational simulation-aided design: Utilize molecular docking and molecular dynamics simulations to predict the binding modes and affinities of monomers with key proteins of the PI3K/AKT pathway (e.g., PI3Kα, mTOR), guiding rational design.
2. Structural modification and delivery optimization: For monomers with low bioavailability or suboptimal selectivity (e.g., curcumin), employ strategies such as prodrug design, glycosylation modification, or advanced nanocarriers (e.g., liposomes, exosomes) to enhance their targeting, solubility, and stability.
3. Natural product-inspired synthesis: Based on validated pharmacophores (e.g., the 5,7-dihydroxy structure of flavonoids), design and synthesize libraries of simplified derivatives with enhanced activity and selectivity through chemical synthesis, overcoming the complexities and low yields associated with natural product extraction.

## 13. Future perspectives

With the deep integration of multi-omics technologies, artificial intelligence, and synthetic biology, research on TCM monomers targeting the PI3K/AKT pathway is poised to enter a new “trinity” phase:

Mechanistic depth: Expanding from single pathway regulation to the systems biology level, encompassing the gut–lung axis, metabolism–immune crosstalk, and cancer stem cells. Techniques like single-cell sequencing and spatial transcriptomics will be employed to analyze the reprogramming effects of monomers on the TME at single-cell resolution.

Structural innovation: Based on in-depth SAR studies and computer-aided drug design, develop a new generation of PI3K/AKT pathway modulators that combine the multi-target synergistic safety profile of natural products with the high efficacy and selectivity of synthetic drugs.

Clinical translation: Closely integrate organoid models, patient-derived xenograft models, and AI-assisted clinical trial design to accurately predict patient response. This will accelerate the advancement of TCM monomers with clearly defined PI3K/AKT pathway regulatory activity and favorable pharmacokinetic properties into Phase I/II clinical trials.

Deepening mechanistic understanding through SAR studies: While this review categorizes TCM monomers based on their primary mechanism of action (e.g., direct pathway inhibition, miRNA modulation) to highlight functional relevance, a deeper layer of understanding awaits exploration. Future research should prioritize SAR investigations to bridge chemical structure with biological specificity. This entails systematically correlating key pharmacophores (e.g., the catechol moiety in flavonoids like quercetin, the quinone core in naphthoquinones like plumbagin and juglone, or the specific glycosylation patterns in flavonoid glycosides) with their binding affinity and inhibitory potency against distinct nodes of the PI3K/AKT pathway (e.g., the ATP-binding pocket of PI3Kα versus the pleckstrin homology domain of AKT). Such SAR studies, facilitated by computational docking, molecular dynamics simulations, and medicinal chemistry, are essential for the rational design of next-generation derivatives that are not only more potent but also exhibit improved selectivity and pharmacokinetic properties, moving beyond the inherent polypharmacology of the parent natural products.

Harnessing the PI3K/AKT–immune checkpoint axis: An underexplored but highly promising avenue is the potential of TCM monomers to modulate the immune checkpoint landscape via PI3K/AKT pathway inhibition. The PI3K/AKT pathway is a known upstream regulator of PD-L1 expression in cancer cells and a key determinant of macrophage polarization (M1 vs M2 phenotype) within the TME. Therefore, the inhibitory effects of the reviewed monomers on this central hub may extend beyond direct cytotoxicity to encompass immunomodulation. Future studies should explicitly investigate whether these compounds can downregulate PD-L1 expression on tumor cells or skew tumor-associated macrophages towards an antitumor M1 phenotype. Confirming such mechanisms would not only deepen our understanding of their antitumor effects but, more importantly, would reveal compelling rationale for combination strategies with immune checkpoint inhibitors. A monomer that concurrently inhibits tumor-intrinsic survival signaling via PI3K/AKT and reverses local immune suppression could potentially re-sensitize “cold” tumors to immunotherapy, addressing a major clinical challenge in NSCLC treatment.

Therapeutic window, selectivity, and comparison with classical inhibitors: The ubiquitous physiological role of the PI3K/AKT pathway necessitates a critical evaluation of the therapeutic window for any modulating agent. While classical ATP-competitive PI3K/AKT inhibitors (e.g., idelalisib, everolimus) are engineered for high target specificity, their clinical application is often hampered by on-target toxicities (e.g., hyperglycemia, rash, hepatotoxicity) due to pathway disruption in normal tissues. In contrast, the TCM monomers reviewed here predominantly exhibit a polypharmacological profile. This multi-target nature could, in theory, offer advantages in overcoming resistance by simultaneously inhibiting complementary pathways, but it inherently raises significant concerns about a more complex and less predictable off-target and systemic toxicity profile. A critical and near-universal gap in the current literature is the lack of systematic, comparative assessment of tumor versus normal tissue selectivity for these compounds. For instance, while vitexin has been reported to show minimal toxicity toward normal bronchial epithelial cells (16HBE) and Icariin toward BEAS-2B cells, such selectivity assessments are anecdotal and absent for the vast majority of monomers. Importantly, the potential immunomodulatory or other systemic effects of these compounds – which could be either beneficial or detrimental – are largely unexplored.

This integrated research paradigm, combining “mechanistic dissection–structural optimization–clinical validation,” not only provides innovative solutions to overcome the heterogeneity and drug resistance of NSCLC but will also vigorously promote the modernization and internationalization of TCM, truly integrating it into the global drug discovery system of precision medicine.

Ultimately, the development of TCM monomers targeting the PI3K/AKT hub should be guided by a biomarker-driven precision medicine framework. This entails not only identifying the direct molecular targets of these compounds but also discovering predictive biomarkers (e.g., specific mutations, expression signatures, or microbiota profiles) that can stratify patients most likely to benefit, thereby mirroring the successful clinical translation pathway of other targeted agents.^[212]^

## Author contributions

**Conceptualization:** Shihu Gan, Mingxing Wang.

**Methodology:** Jipu Pan, Mingxing Wang.

**Resources:** Shanjun Yang.

**Software:** Jipu Pan.

**Writing – original draft:** Shihu Gan.

**Writing – review & editing:** Shihu Gan, Shanjun Yang.

## References

[1] Wang Y Yu F Qiu F Fang K. Global burden of respiratory system cancers in 2022 and 2050: incidence and mortality estimates from GLOBOCAN. BMC Cancer. 2025;25:1832. 41316070 10.1186/s12885-025-15209-2 PMC12661822

[2] Ferlay J Colombet M Soerjomataram I. Cancer statistics for the year 2020: an overview. Int J Cancer. 2021;149:778–89. 10.1002/ijc.33588 33818764

[3] Yousefi M Bahrami T Salmaninejad A Nosrati R Ghaffari P Ghaffari SH. Lung cancer-associated brain metastasis: molecular mechanisms and therapeutic options. Cell Oncol (Dordr). 2017;40:419–41. 28921309 10.1007/s13402-017-0345-5 PMC13001560

[4] Henschke CI Yip R Shaham D. A 20-year follow-up of the international Early Lung Cancer Action Program (I-ELCAP). Radiology. 2023;309:e231988. 37934099 10.1148/radiol.231988 PMC10698500

[5] Forde PM Spicer JD Provencio M. Overall survival with neoadjuvant nivolumab plus chemotherapy in lung cancer. N Engl J Med. 2025;393:741–52. 40454642 10.1056/NEJMoa2502931

[6] Chang JY Lin SH Dong W. Stereotactic ablative radiotherapy with or without immunotherapy for early-stage or isolated lung parenchymal recurrent node-negative non-small-cell lung cancer: an open-label, randomised, phase 2 trial. Lancet. 2023;402:871–81. 37478883 10.1016/S0140-6736(23)01384-3 PMC10529504

[7] Burdett S Pignon JP Tierney J. Adjuvant chemotherapy for resected early-stage non-small cell lung cancer. Cochrane Database Syst Rev. 2015;2015:CD011430. 25730344 10.1002/14651858.CD011430 PMC10542092

[8] Mitsudomi T Heymach JV Reck M. Surgical outcomes with neoadjuvant durvalumab plus chemotherapy followed by adjuvant durvalumab in resectable NSCLC. J Thorac Oncol. 2025;20:1639–54. 40545237 10.1016/j.jtho.2025.06.015

[9] Biomarkers Definitions Working Group. Biomarkers and surrogate endpoints: preferred definitions and conceptual framework. Clin Pharmacol Ther. 2001;69:89–95. 11240971 10.1067/mcp.2001.113989

[10] Gomes M Teixeira AL Coelho A Araújo A Medeiros R. The role of inflammation in lung cancer. Adv Exp Med Biol. 2014;816:1–23. 24818717 10.1007/978-3-0348-0837-8_1

[11] Coussens LM Werb Z. Inflammation and cancer. Nature. 2002;420:860–7. 12490959 10.1038/nature01322 PMC2803035

[12] DeNardo DG Ruffell B. Macrophages as regulators of tumour immunity and immunotherapy. Nat Rev Immunol. 2019;19:369–82. 30718830 10.1038/s41577-019-0127-6 PMC7339861

[13] Zaman R Epelman S. Resident cardiac macrophages: heterogeneity and function in health and disease. Immunity. 2022;55:1549–63. 36103852 10.1016/j.immuni.2022.08.009

[14] Bögels M Braster R Nijland PG. Carcinoma origin dictates differential skewing of monocyte function. Oncoimmunology. 2012;1:798–809. 23162747 10.4161/onci.20427 PMC3489735

[15] Pan Y Yu Y Wang X Zhang T. Tumor-associated macrophages in tumor immunity. Front Immunol. 2020;11:583084. 33365025 10.3389/fimmu.2020.583084 PMC7751482

[16] Mantovani A Sica A Sozzani S Allavena P Vecchi A Locati M. The chemokine system in diverse forms of macrophage activation and polarization. Trends Immunol. 2004;25:677–86. 15530839 10.1016/j.it.2004.09.015

[17] Fridlender ZG Sun J Kim S. Polarization of tumor-associated neutrophil phenotype by TGF-beta: “N1” versus “N2” TAN. Cancer Cell. 2009;16:183–94. 19732719 10.1016/j.ccr.2009.06.017 PMC2754404

[18] Güngör N Knaapen AM Munnia A. Genotoxic effects of neutrophils and hypochlorous acid. Mutagenesis. 2010;25:149–54. 19892774 10.1093/mutage/gep053

[19] Coussens LM Tinkle CL Hanahan D Werb Z. MMP-9 supplied by bone marrow-derived cells contributes to skin carcinogenesis. Cell. 2000;103:481–90. 11081634 10.1016/s0092-8674(00)00139-2 PMC2843102

[20] Zhang X Kohli M Zhou Q Graves DT Amar S. Short- and long-term effects of IL-1 and TNF antagonists on periodontal wound healing. J Immunol. 2004;173:3514–23. 15322216 10.4049/jimmunol.173.5.3514

[21] Song J Feng L Zhong R. Icariside II inhibits the EMT of NSCLC cells in inflammatory microenvironment via down-regulation of Akt/NF-κB signaling pathway. Mol Carcinog. 2017;56:36–48. 26859114 10.1002/mc.22471

[22] Shimizu M Tanaka N. IL-8-induced O-GlcNAc modification via GLUT3 and GFAT regulates cancer stem cell-like properties in colon and lung cancer cells. Oncogene. 2019;38:1520–33. 30305725 10.1038/s41388-018-0533-4

[23] Lei Y Chen T Li Y. O-GlcNAcylation of PFKFB3 is required for tumor cell proliferation under hypoxia. Oncogenesis. 2020;9:21. 32060258 10.1038/s41389-020-0208-1 PMC7021673

[24] Liu CC Lin JH Hsu TW. IL-6 enriched lung cancer stem-like cell population by inhibition of cell cycle regulators via DNMT1 upregulation. Int J Cancer. 2015;136:547–59. 24947242 10.1002/ijc.29033

[25] Ben-Baruch A. Inflammation-associated immune suppression in cancer: the roles played by cytokines, chemokines and additional mediators. Semin Cancer Biol. 2006;16:38–52. 16139507 10.1016/j.semcancer.2005.07.006

[26] Greenburg G Hay ED. Epithelia suspended in collagen gels can lose polarity and express characteristics of migrating mesenchymal cells. J Cell Biol. 1982;95:333–9. 7142291 10.1083/jcb.95.1.333 PMC2112361

[27] Otsuki Y Saya H Arima Y. Prospects for new lung cancer treatments that target EMT signaling. Dev Dyn. 2018;247:462–72. 28960588 10.1002/dvdy.24596

[28] Lee HW Jose CC Cuddapah S. Epithelial-mesenchymal transition: insights into nickel-induced lung diseases. Semin Cancer Biol. 2021;76:99–109. 34058338 10.1016/j.semcancer.2021.05.020 PMC8627926

[29] Lu W Kang Y. Epithelial-mesenchymal plasticity in cancer progression and metastasis. Dev Cell. 2019;49:361–74. 31063755 10.1016/j.devcel.2019.04.010 PMC6506183

[30] Huang L Liu X Chen Q. TGF-β-induced lncRNA TBUR1 promotes EMT and metastasis in lung adenocarcinoma via hnRNPC-mediated GRB2 mRNA stabilization. Cancer Lett. 2024;600:217153. 39102940 10.1016/j.canlet.2024.217153

[31] Derynck R Budi EH. Specificity, versatility, and control of TGF-β family signaling. Sci Signal. 2019;12:eaav5183. 30808818 10.1126/scisignal.aav5183 PMC6800142

[32] Khan AQ Hasan A Mir SS Rashid K Uddin S Steinhoff M. Exploiting transcription factors to target EMT and cancer stem cells for tumor modulation and therapy. Semin Cancer Biol. 2024;100:1–16. 38503384 10.1016/j.semcancer.2024.03.002

[33] Lai CY Yeh DW Lu CH. Epigenetic silencing of ubiquitin specific protease 4 by Snail1 contributes to macrophage-dependent inflammation and therapeutic resistance in lung cancer. Cancers (Basel). 2020;12:148. 31936290 10.3390/cancers12010148 PMC7016945

[34] Kaufhold S Bonavida B. Central role of Snail1 in the regulation of EMT and resistance in cancer: a target for therapeutic intervention. J Exp Clin Cancer Res. 2014;33:62. 25084828 10.1186/s13046-014-0062-0 PMC4237825

[35] Yang B Zhang W Zhang M Wang X Peng S Zhang R. KRT6A promotes EMT and cancer stem cell transformation in lung adenocarcinoma. Technol Cancer Res Treat. 2020;19:1533033820921248. 32329414 10.1177/1533033820921248 PMC7225834

[36] Tiwari N Gheldof A Tatari M Christofori G. EMT as the ultimate survival mechanism of cancer cells. Semin Cancer Biol. 2012;22:194–207. 22406545 10.1016/j.semcancer.2012.02.013

[37] Sies H Jones DP. Reactive oxygen species (ROS) as pleiotropic physiological signalling agents. Nat Rev Mol Cell Biol. 2020;21:363–83. 32231263 10.1038/s41580-020-0230-3

[38] Park HS Kim SR Lee YC. Impact of oxidative stress on lung diseases. Respirology. 2009;14:27–38. 19144046 10.1111/j.1440-1843.2008.01447.x

[39] Saleh EAM Al-Dolaimy F Almajidi YQ. Oxidative stress affects the beginning of the growth of cancer cells through a variety of routes. Pathol Res Pract. 2023;249:154664. 37573621 10.1016/j.prp.2023.154664

[40] Filaire E Dupuis C Galvaing G. Lung cancer: what are the links with oxidative stress, physical activity and nutrition. Lung Cancer. 2013;82:383–9. 24161719 10.1016/j.lungcan.2013.09.009

[41] Weng MS Chang JH Hung WY Yang YC Chien MH. The interplay of reactive oxygen species and the epidermal growth factor receptor in tumor progression and drug resistance. J Exp Clin Cancer Res. 2018;37:61. 29548337 10.1186/s13046-018-0728-0 PMC5857086

[42] Lee J Roh JL. Targeting GPX4 in human cancer: implications of ferroptosis induction for tackling cancer resilience. Cancer Lett. 2023;559:216119. 36893895 10.1016/j.canlet.2023.216119

[43] Wei J Zhu L. The role of glutathione peroxidase 4 in the progression, drug resistance, and targeted therapy of non-small cell lung cancer. Oncol Res. 2025;33:863–72. 40191731 10.32604/or.2024.054201 PMC11964886

[44] Riondino S Rosenfeld R Formica V. Effectiveness of immunotherapy in non-small cell lung cancer patients with a diagnosis of COPD: is this a hidden prognosticator for survival and a risk factor for immune-related adverse events? Cancers (Basel). 2024;16:1251. 38610929 10.3390/cancers16071251 PMC11011072

[45] Dahabieh MS DeCamp LM Oswald BM. The prostacyclin receptor PTGIR is a NRF2-dependent regulator of CD8⁺ T cell exhaustion. Nat Immunol. 2025:1139–51. 40579556 10.1038/s41590-025-02185-9 PMC12208871

[46] Zhou S Liu R Yuan K. Proteomics analysis of tumor microenvironment: implications of metabolic and oxidative stresses in tumorigenesis. Mass Spectrom Rev. 2013;32:267–311. 23165949 10.1002/mas.21362

[47] Levy JMM Towers CG Thorburn A. Targeting autophagy in cancer. Nat Rev Cancer. 2017;17:528–42. 28751651 10.1038/nrc.2017.53 PMC5975367

[48] Lu Y Li Z Zhang S Zhang T Liu Y Zhang L. Cellular mitophagy: mechanism, roles in diseases and small molecule pharmacological regulation. Theranostics. 2023;13:736–66. 36632220 10.7150/thno.79876 PMC9830443

[49] Zhang J Tripathi DN Jing J. ATM functions at the peroxisome to induce pexophagy in response to ROS. Nat Cell Biol. 2015;17:1259–69. 26344566 10.1038/ncb3230 PMC4589490

[50] Schuck S Gallagher CM Walter P. ER-phagy mediates selective degradation of endoplasmic reticulum independently of the core autophagy machinery. J Cell Sci. 2014;127(Pt 18):4078–88. 25052096 10.1242/jcs.154716 PMC4163648

[51] Mochida K Oikawa Y Kimura Y. Receptor-mediated selective autophagy degrades the endoplasmic reticulum and the nucleus. Nature. 2015;522:359–62. 26040717 10.1038/nature14506

[52] Trejo-Solís C Serrano-Garcia N Escamilla-Ramírez A. Autophagic and apoptotic pathways as targets for chemotherapy in glioblastoma. Int J Mol Sci. 2018;19:3773. 30486451 10.3390/ijms19123773 PMC6320836

[53] Gatica D Klionsky DJ. New tricks of an old autophagy regulator: AMPK-dependent regulation of autophagy through CCNY (cyclin Y)-CDK16. Autophagy. 2020;16:973–4. 32401167 10.1080/15548627.2020.1756665 PMC7469459

[54] Wang X Li S Lin S. Oncogenic RAS induces a distinctive form of non-canonical autophagy mediated by the P38–ULK1–PI4KB axis. Cell Res. 2025;35:399–422. 40055523 10.1038/s41422-025-01085-9 PMC12134136

[55] Kim YC Guan KL. mTOR: a pharmacologic target for autophagy regulation. J Clin Invest. 2015;125:25–32. 25654547 10.1172/JCI73939 PMC4382265

[56] Deleyto-Seldas N Efeyan A. The mTOR–autophagy axis and the control of metabolism. Front Cell Dev Biol. 2021;9:655731. 34277603 10.3389/fcell.2021.655731 PMC8281972

[57] O’Neill EJ Sze NSK MacPherson REK Tsiani E. Carnosic acid against lung cancer: induction of autophagy and activation of sestrin-2/LKB1/AMPK signalling. Int J Mol Sci. 2024;25:1950. 38396629 10.3390/ijms25041950 PMC10888478

[58] You L Wang Z Li H. The role of STAT3 in autophagy. Autophagy. 2015;11:729–39. 25951043 10.1080/15548627.2015.1017192 PMC4509450

[59] Shi B Ma M Zheng Y Pan Y Lin X. mTOR and Beclin1: two key autophagy-related molecules and their roles in myocardial ischemia/reperfusion injury. J Cell Physiol. 2019;234:12562–8. 30618070 10.1002/jcp.28125 PMC13484135

[60] Li X He S Ma B. Autophagy and autophagy-related proteins in cancer. Mol Cancer. 2020;19:12. 31969156 10.1186/s12943-020-1138-4 PMC6975070

[61] Qu X Yu J Bhagat G. Promotion of tumorigenesis by heterozygous disruption of the beclin 1 autophagy gene. J Clin Invest. 2003;112:1809–20. 14638851 10.1172/JCI20039 PMC297002

[62] Guo JY Chen HY Mathew R. Activated Ras requires autophagy to maintain oxidative metabolism and tumorigenesis. Genes Dev. 2011;25:460–70. 21317241 10.1101/gad.2016311 PMC3049287

[63] Tittarelli A Janji B Van Moer K Noman MZ Chouaib S. The selective degradation of synaptic connexin 43 protein by hypoxia-induced autophagy impairs natural killer cell-mediated tumor cell killing. J Biol Chem. 2015;290:23670–9. 26221040 10.1074/jbc.M115.651547 PMC4583044

[64] Olejarz W Łacheta D Kubiak-Tomaszewska G. Matrix metalloproteinases as biomarkers of atherosclerotic plaque instability. Int J Mol Sci. 2020;21:3946. 32486345 10.3390/ijms21113946 PMC7313469

[65] Riihilä P Nissinen L Kähäri VM. Matrix metalloproteinases in keratinocyte carcinomas. Exp Dermatol. 2021;30:50–61. 32869366 10.1111/exd.14183 PMC7821196

[66] de Almeida LGN Thode H Eslambolchi Y. Matrix metalloproteinases: from molecular mechanisms to physiology, pathophysiology, and pharmacology. Pharmacol Rev. 2022;74:712–68. 35738680 10.1124/pharmrev.121.000349

[67] Zhang H Zhao B Zhai ZG Zheng JD Wang YK Zhao YY. Expression and clinical significance of MMP-9 and P53 in lung cancer. Eur Rev Med Pharmacol Sci. 2021;25:1358–65. 33629306 10.26355/eurrev_202102_24844

[68] Jafarian AH Forooshani MK Reisi H Roshan NM. Matrix metalloproteinase-9 (MMP-9) expression in non-small cell lung carcinoma and its association with clinicopathologic factors. Iranian J Pathol. 2020;15:326–33. 10.30699/ijp.2020.95177.1940 PMC7477682 32944046

[69] Bergers G Brekken R McMahon G. Matrix metalloproteinase-9 triggers the angiogenic switch during carcinogenesis. Nat Cell Biol. 2000;2:737–44. 11025665 10.1038/35036374 PMC2852586

[70] Deryugina EI Quigley JP. Tumor angiogenesis: MMP-mediated induction of intravasation- and metastasis-sustaining neovasculature. Matrix Biol. 2015;44-46:94–112. 25912949 10.1016/j.matbio.2015.04.004 PMC5079283

[71] Zhang XS Wang KY Gao JQ Li RJ Guan QB Song L. Study on the expression of p53 and MMP-2 in patients with lung cancer after interventional therapy. Oncol Lett. 2018;16:4291–6. 30214563 10.3892/ol.2018.9185 PMC6126205

[72] Chen W Xia T Wang D. Human astrocytes secrete IL-6 to promote glioma migration and invasion through upregulation of cytomembrane MMP14. Oncotarget. 2016;7:62425–38. 27613828 10.18632/oncotarget.11515 PMC5308737

[73] Stawowczyk M Wellenstein MD Lee SB. Matrix Metalloproteinase 14 promotes lung cancer by cleavage of Heparin-Binding EGF-like Growth Factor. Neoplasia. 2017;19:55–64. 28013056 10.1016/j.neo.2016.11.005 PMC5198728

[74] Illman SA Lehti K Keski-Oja J Lohi J. Epilysin (MMP-28) induces TGF-beta mediated epithelial to mesenchymal transition in lung carcinoma cells. J Cell Sci. 2006;119(Pt 18):3856–65. 16940349 10.1242/jcs.03157

[75] Ma B Ran R Liao HY Zhang HH. The paradoxical role of matrix metalloproteinase-11 in cancer. Biomed Pharmacother. 2021;141:111899. 34346316 10.1016/j.biopha.2021.111899

[76] Gill N Wlodarska M Finlay BB. The future of mucosal immunology: studying an integrated system-wide organ. Nat Immunol. 2010;11:558–60. 20562837 10.1038/ni0710-558

[77] Khan FH Bhat BA Sheikh BA. Microbiome dysbiosis and epigenetic modulations in lung cancer: from pathogenesis to therapy. Semin Cancer Biol. 2022;86(Pt 3):732–42. 34273520 10.1016/j.semcancer.2021.07.005

[78] Hong T Wang R Wang X. Interplay between the intestinal microbiota and acute graft-versus-host disease: experimental evidence and clinical significance. Front Immunol. 2021;12:644982. 33815399 10.3389/fimmu.2021.644982 PMC8010685

[79] Bhatt AP Redinbo MR Bultman SJ. The role of the microbiome in cancer development and therapy. CA Cancer J Clin. 2017;67:326–44. 28481406 10.3322/caac.21398 PMC5530583

[80] Bingula R Filaire M Radosevic-Robin N. Desired turbulence? Gut–lung axis, immunity, and lung cancer. J Oncol. 2017;2017:5035371. 29075294 10.1155/2017/5035371 PMC5623803

[81] Kho ZY Lal SK. The human gut microbiome - A potential controller of wellness and disease. Front Microbiol. 2018;9:1835. 30154767 10.3389/fmicb.2018.01835 PMC6102370

[82] Garrett WS. Cancer and the microbiota. Science. 2015;348:80–6. 25838377 10.1126/science.aaa4972 PMC5535753

[83] Zhuang H Cheng L Wang Y. Dysbiosis of the gut microbiome in lung cancer. Front Cell Infect Microbiol. 2019;9:112. 31065547 10.3389/fcimb.2019.00112 PMC6489541

[84] Preet R Islam MA Shim J. Gut commensal Bifidobacterium-derived extracellular vesicles modulate the therapeutic effects of anti-PD-1 in lung cancer. Nat Commun. 2025;16:3500. 40221398 10.1038/s41467-025-58553-4 PMC11993705

[85] Pizzo F Maroccia Z Ferri IH Fiorentini C. Role of the microbiota in lung cancer: insights on prevention and treatment. Int J Mol Sci. 2022;23:6138. 35682816 10.3390/ijms23116138 PMC9181592

[86] Derosa L Routy B Thomas AM. Intestinal Akkermansia muciniphila predicts clinical response to PD-1 blockade in patients with advanced non-small-cell lung cancer. Nat Med. 2022;28:315–24. 35115705 10.1038/s41591-021-01655-5 PMC9330544

[87] Chen J Yu X Wu X Chai K Wang S. Causal relationships between gut microbiota, immune cell, and non-small cell lung cancer: a two-step, two-sample Mendelian randomization study. J Cancer. 2024;15:1890–7. 38434967 10.7150/jca.92699 PMC10905411

[88] Tomita Y Goto Y Sakata S. Clostridium butyricum therapy restores the decreased efficacy of immune checkpoint blockade in lung cancer patients receiving proton pump inhibitors. Oncoimmunology. 2022;11:2081010. 35655708 10.1080/2162402X.2022.2081010 PMC9154751

[89] Botticelli A Vernocchi P Marini F. Gut metabolomics profiling of non-small cell lung cancer (NSCLC) patients under immunotherapy treatment. J Transl Med. 2020;18:49. 32014010 10.1186/s12967-020-02231-0 PMC6998840

[90] Vernocchi P Gili T Conte F. Network analysis of gut microbiome and metabolome to discover microbiota-linked biomarkers in patients affected by non-small cell lung cancer. Int J Mol Sci. 2020;21:8730. 33227982 10.3390/ijms21228730 PMC7699235

[91] Gui Q Li H Wang A. The association between gut butyrate-producing bacteria and non-small-cell lung cancer. J Clin Lab Anal. 2020;34:e23318. 32227387 10.1002/jcla.23318 PMC7439349

[92] Zheng Y Fang Z Xue Y. Specific gut microbiome signature predicts the early-stage lung cancer. Gut Microbes. 2020;11:1030–42. 32240032 10.1080/19490976.2020.1737487 PMC7524275

[93] Liu Z Wang C Lin C. Pyroptosis as a double-edged sword: the pathogenic and therapeutic roles in inflammatory diseases and cancers. Life Sci. 2023;318:121498. 36780939 10.1016/j.lfs.2023.121498

[94] Liang X Qin Y Wu D Wang Q Wu H. Pyroptosis: a double-edged sword in lung cancer and other respiratory diseases. Cell Commun Signal. 2024;22:40. 38225586 10.1186/s12964-023-01458-w PMC10790448

[95] Chen Y Sun L Liu H Li J Guo L Wang Z. KLF4 interacts with TXNIP to modulate the pyroptosis in ulcerative colitis via regulating NLRP3 signaling. Immun Inflamm Dis. 2024;12:e1199. 38411328 10.1002/iid3.1199 PMC10898204

[96] Li H Yang T Zhang J. Pyroptotic cell death: an emerging therapeutic opportunity for radiotherapy. Cell Death Discov. 2024;10:32. 38228635 10.1038/s41420-024-01802-0 PMC10791972

[97] Broz P Dixit VM. Inflammasomes: mechanism of assembly, regulation and signalling. Nat Rev Immunol. 2016;16:407–20. 27291964 10.1038/nri.2016.58

[98] Zhang P Liu Y Hu L. NLRC4 inflammasome-dependent cell death occurs by a complementary series of three death pathways and determines lethality in mice. Sci Adv. 2021;7:eabi9471. 34678072 10.1126/sciadv.abi9471 PMC8535822

[99] Shi X Sun Q Hou Y. Recognition and maturation of IL-18 by caspase-4 noncanonical inflammasome. Nature. 2023;624:442–50. 37993714 10.1038/s41586-023-06742-w

[100] Wei C Jiang W Wang R. Brain endothelial GSDMD activation mediates inflammatory BBB breakdown. Nature. 2024;629:893–900. 38632402 10.1038/s41586-024-07314-2

[101] Shi J Zhao Y Wang K. Cleavage of GSDMD by inflammatory caspases determines pyroptotic cell death. Nature. 2015;526:660–5. 26375003 10.1038/nature15514

[102] Eren E Özören N. The NLRP3 inflammasome: a new player in neurological diseases. Turk J Biol. 2019;43:349–59. 31892810 10.3906/biy-1909-31 PMC6911260

[103] Li L Song D Qi L. Photodynamic therapy induces human esophageal carcinoma cell pyroptosis by targeting the PKM2/caspase-8/caspase-3/GSDME axis. Cancer Lett. 2021;520:143–59. 34256094 10.1016/j.canlet.2021.07.014

[104] Qiao L Zhu G Jiang T. Self-destructive copper carriers induce pyroptosis and cuproptosis for efficient tumor immunotherapy against dormant and recurrent tumors. Adv Mater. 2024;36:e2308241. 37820717 10.1002/adma.202308241

[105] Wei T Zhang C Song Y. Molecular mechanisms and roles of pyroptosis in acute lung injury. Chin Med J (Engl). 2022;135:2417–26. 36583860 10.1097/CM9.0000000000002425 PMC9945565

[106] Setrerrahmane S Xu H. Tumor-related interleukins: old validated targets for new anti-cancer drug development. Mol Cancer. 2017;16:153. 28927416 10.1186/s12943-017-0721-9 PMC5606116

[107] Bera A Ghosh-Choudhury N Dey N. NFκB-mediated cyclin D1 expression by microRNA-21 influences renal cancer cell proliferation. Cell Signal. 2013;25:2575–86. 23981302 10.1016/j.cellsig.2013.08.005 PMC3896302

[108] Zhang Y Jia Q Li J. Copper-bacteriochlorin nanosheet as a specific pyroptosis inducer for robust tumor immunotherapy. Adv Mater. 2023;35:e2305073. 37421648 10.1002/adma.202305073

[109] Elhanani O Ben-Uri R Keren L. Spatial profiling technologies illuminate the tumor microenvironment. Cancer Cell. 2023;41:404–20. 36800999 10.1016/j.ccell.2023.01.010

[110] Kao KC Vilbois S Tsai CH Ho PC. Metabolic communication in the tumour-immune microenvironment. Nat Cell Biol. 2022;24:1574–83. 36229606 10.1038/s41556-022-01002-x

[111] Desbois M Wang Y. Cancer-associated fibroblasts: key players in shaping the tumor immune microenvironment. Immunol Rev. 2021;302:241–58. 34075584 10.1111/imr.12982

[112] Corsa CA Brenot A Grither WR. The action of discoidin domain receptor 2 in basal tumor cells and stromal cancer-associated fibroblasts is critical for breast cancer metastasis. Cell Rep. 2016;15:2510–23. 27264173 10.1016/j.celrep.2016.05.033 PMC4909540

[113] Huang J Zhang L Wan D. Extracellular matrix and its therapeutic potential for cancer treatment. Signal Transduct Target Ther. 2021;6:153. 33888679 10.1038/s41392-021-00544-0 PMC8062524

[114] Lu C Liu Y Ali NM Zhang B Cui X. The role of innate immune cells in the tumor microenvironment and research progress in anti-tumor therapy. Front Immunol. 2022;13:1039260. 36741415 10.3389/fimmu.2022.1039260 PMC9893925

[115] Kolahian S Öz HH Zhou B Griessinger CM Rieber N Hartl D. The emerging role of myeloid-derived suppressor cells in lung diseases. Eur Respir J. 2016;47:967–77. 26846830 10.1183/13993003.01572-2015

[116] Qu X Zhuang G Yu L Meng G Ferrara N. Induction of Bv8 expression by granulocyte colony-stimulating factor in CD11b+Gr1+ cells: key role of Stat3 signaling. J Biol Chem. 2012;287:19574–84. 22528488 10.1074/jbc.M111.326801 PMC3365993

[117] Hu P Shen M Zhang P. Intratumoral neutrophil granulocytes contribute to epithelial-mesenchymal transition in lung adenocarcinoma cells. Tumour Biol. 2015;36:7789–96. 25944163 10.1007/s13277-015-3484-1

[118] Joffre O Nolte MA Spörri R Reis e Sousa C. Inflammatory signals in dendritic cell activation and the induction of adaptive immunity. Immunol Rev. 2009;227:234–47. 19120488 10.1111/j.1600-065X.2008.00718.x

[119] Albert ML Sauter B Bhardwaj N. Dendritic cells acquire antigen from apoptotic cells and induce class I-restricted CTLs. Nature. 1998;392:86–9. 9510252 10.1038/32183

[120] Smyth MJ Cretney E Kelly JM. Activation of NK cell cytotoxicity. Mol Immunol. 2005;42:501–10. 15607806 10.1016/j.molimm.2004.07.034

[121] Mansouri S Heylmann D Stiewe T Kracht M Savai R. Cancer genome and tumor microenvironment: reciprocal crosstalk shapes lung cancer plasticity. Elife. 2022;11:e79895. 36074553 10.7554/eLife.79895 PMC9457687

[122] Genova C Rijavec E Grossi F. Tumor microenvironment as a potential source of clinical biomarkers in non-small cell lung cancer: can we use enemy territory at our advantage? J Thorac Dis. 2017;9:4300–4. 29268496 10.21037/jtd.2017.10.66 PMC5721017

[123] Wu J Li L Zhang H. A risk model developed based on tumor microenvironment predicts overall survival and associates with tumor immunity of patients with lung adenocarcinoma. Oncogene. 2021;40:4413–24. 34108619 10.1038/s41388-021-01853-y

[124] Xing X Yang F Huang Q. Decoding the multicellular ecosystem of lung adenocarcinoma manifested as pulmonary subsolid nodules by single-cell RNA sequencing. Sci Adv. 2021;7:eabd9738. 33571124 10.1126/sciadv.abd9738 PMC7840134

[125] Huang C Li H Xu Y. BICC1 drives pancreatic cancer progression by inducing VEGF-independent angiogenesis. Signal Transduct Target Ther. 2023;8:271. 37443111 10.1038/s41392-023-01478-5 PMC10344882

[126] Liu ZL Chen HH Zheng LL Sun LP Shi L. Angiogenic signaling pathways and anti-angiogenic therapy for cancer. Signal Transduct Target Ther. 2023;8:198. 37169756 10.1038/s41392-023-01460-1 PMC10175505

[127] Guo GX Qiu YH Liu Y. Fucoxanthin attenuates angiogenesis by blocking the VEGFR2-mediated signaling pathway through binding the vascular endothelial growth factor. J Agric Food Chem. 2024;72:21610–23. 39292861 10.1021/acs.jafc.4c05464

[128] Yasui M Yamamoto H Ngan CY. Antisense to cyclin D1 inhibits vascular endothelial growth factor-stimulated growth of vascular endothelial cells: implication of tumor vascularization. Clin Cancer Res. 2006;12:4720–9. 16899623 10.1158/1078-0432.CCR-05-1213

[129] Morales-Ruiz M Fulton D Sowa G. Vascular endothelial growth factor-stimulated actin reorganization and migration of endothelial cells is regulated via the serine/threonine kinase Akt. Circ Res. 2000;86:892–6. 10785512 10.1161/01.res.86.8.892

[130] Rousseau S Houle F Kotanides H. Vascular endothelial growth factor (VEGF)-driven actin-based motility is mediated by VEGFR2 and requires concerted activation of stress-activated protein kinase 2 (SAPK2/p38) and geldanamycin-sensitive phosphorylation of focal adhesion kinase. J Biol Chem. 2000;275:10661–72. 10744763 10.1074/jbc.275.14.10661

[131] Funahashi Y Shawber CJ Sharma A Kanamaru E Choi YK Kitajewski J. Notch modulates VEGF action in endothelial cells by inducing Matrix Metalloprotease activity. Vasc Cell. 2011;3:2. 21349159 10.1186/2045-824X-3-2 PMC3039832

[132] Avraham HK Lee TH Koh Y. Vascular endothelial growth factor regulates focal adhesion assembly in human brain microvascular endothelial cells through activation of the focal adhesion kinase and related adhesion focal tyrosine kinase. J Biol Chem. 2003;278:36661–8. 12844492 10.1074/jbc.M301253200

[133] Mallat Z Tedgui A. Apoptosis in the vasculature: mechanisms and functional importance. Br J Pharmacol. 2000;130:947–62. 10882378 10.1038/sj.bjp.0703407 PMC1572165

[134] He H Venema VJ Gu X Venema RC Marrero MB Caldwell RB. Vascular endothelial growth factor signals endothelial cell production of nitric oxide and prostacyclin through flk-1/KDR activation of c-Src. J Biol Chem. 1999;274:25130–5. 10455194 10.1074/jbc.274.35.25130

[135] Huang L Wang F Wang Y. Acidic fibroblast growth factor promotes endothelial progenitor cells function via Akt/FOXO3a Pathway. PLoS One. 2015;10:e0129665. 26061278 10.1371/journal.pone.0129665 PMC4463857

[136] Jia T Jacquet T Dalonneau F. FGF-2 promotes angiogenesis through a SRSF1/SRSF3/SRPK1-dependent axis that controls VEGFR1 splicing in endothelial cells. BMC Biol. 2021;19:173. 34433435 10.1186/s12915-021-01103-3 PMC8390225

[137] Pan X Li X Dong L. Tumour vasculature at single-cell resolution. Nature. 2024;632:429–36. 38987599 10.1038/s41586-024-07698-1

[138] Axnick J Lammert E. Vascular lumen formation. Curr Opin Hematol. 2012;19:192–8. 22488306 10.1097/MOH.0b013e3283523ebc

[139] Li Y Zhao L Li XF. Hypoxia and the tumor microenvironment. Technol Cancer Res Treat. 2021;20:15330338211036304. 34350796 10.1177/15330338211036304 PMC8358492

[140] Carmeliet P Jain RK. Principles and mechanisms of vessel normalization for cancer and other angiogenic diseases. Nat Rev Drug Discov. 2011;10:417–27. 21629292 10.1038/nrd3455

[141] Semenza GL. Hypoxia-inducible factor 1: oxygen homeostasis and disease pathophysiology. Trends Mol Med. 2001;7:345–50. 11516994 10.1016/s1471-4914(01)02090-1

[142] Wang K Wang B Wang Z Yang R. Alginic acid inhibits non-small cell lung cancer-induced angiogenesis via activating miR-506 expression. J Nat Med. 2021;75:553–64. 33666835 10.1007/s11418-021-01493-2

[143] Sun J Xiong Y Jiang K. Hypoxia-sensitive long noncoding RNA CASC15 promotes lung tumorigenesis by regulating the SOX4/β-catenin axis. J Exp Clin Cancer Res. 2021;40:12. 33407675 10.1186/s13046-020-01806-5 PMC7789733

[144] Wen T Zhang X Gao Y Tian H Fan L Yang P. SOX4–BMI1 axis promotes non-small cell lung cancer progression and facilitates angiogenesis by suppressing ZNF24. Cell Death Dis. 2024;15:698. 39349443 10.1038/s41419-024-07075-w PMC11442842

[145] Wu Q You L Nepovimova E. Hypoxia-inducible factors: master regulators of hypoxic tumor immune escape. J Hematol Oncol. 2022;15:77. 35659268 10.1186/s13045-022-01292-6 PMC9166526

[146] Seliger B. Molecular mechanisms of HLA class I-mediated immune evasion of human tumors and their role in resistance to immunotherapies. HLA. 2016;88:213–20. 27659281 10.1111/tan.12898

[147] Rosenthal R Cadieux EL Salgado R. Neoantigen-directed immune escape in lung cancer evolution. Nature. 2019;567:479–85. 30894752 10.1038/s41586-019-1032-7 PMC6954100

[148] Chabanon RM Muirhead G Krastev DB. PARP inhibition enhances tumor cell-intrinsic immunity in ERCC1-deficient non-small cell lung cancer. J Clin Invest. 2019;129:1211–28. 30589644 10.1172/JCI123319 PMC6391116

[149] Menter T Tzankov A. Mechanisms of immune evasion and immune modulation by lymphoma cells. Front Oncol. 2018;8:54. 29564225 10.3389/fonc.2018.00054 PMC5845888

[150] Vito A El-Sayes N Mossman K. Hypoxia-driven immune escape in the tumor microenvironment. Cells. 2020;9:992. 32316260 10.3390/cells9040992 PMC7227025

[151] Xu Y Yan J Tao Y. Pituitary hormone α-MSH promotes tumor-induced myelopoiesis and immunosuppression. Science. 2022;377:1085–91. 35926007 10.1126/science.abj2674

[152] Liu H Kuang X Zhang Y. ADORA1 inhibition promotes tumor immune evasion by regulating the ATF3–PD-L1 axis. Cancer Cell. 2020;37:324–39.e8. 32183950 10.1016/j.ccell.2020.02.006

[153] Chand Dakal T Dhabhai B Agarwal D. Mechanistic basis of co-stimulatory CD40–CD40L ligation mediated regulation of immune responses in cancer and autoimmune disorders. Immunobiology. 2020;225:151899. 31899051 10.1016/j.imbio.2019.151899

[154] He D Wang D Lu P. Single-cell RNA sequencing reveals heterogeneous tumor and immune cell populations in early-stage lung adenocarcinomas harboring EGFR mutations. Oncogene. 2021;40:355–68. 33144684 10.1038/s41388-020-01528-0 PMC7808940

[155] Wang M Xiao Y Miao J. Oxidative stress and inflammation: drivers of tumorigenesis and therapeutic opportunities. Antioxidants (Basel). 2025;14:735. 40563367 10.3390/antiox14060735 PMC12189506

[156] Zhang X Ma H Gao Y. The tumor microenvironment: signal transduction. Biomolecules. 2024;14:438. 38672455 10.3390/biom14040438 PMC11048169

[157] Aiello NM Kang Y. Context-dependent EMT programs in cancer metastasis. J Exp Med. 2019;216:1016–26. 30975895 10.1084/jem.20181827 PMC6504222

[158] Yu P Wang HY Tian M. Eukaryotic elongation factor-2 kinase regulates the cross-talk between autophagy and pyroptosis in doxorubicin-treated human melanoma cells in vitro. Acta Pharmacol Sin. 2019;40:1237–44. 30914761 10.1038/s41401-019-0222-z PMC6786479

[159] Niland S Riscanevo AX Eble JA. Matrix metalloproteinases shape the tumor microenvironment in cancer progression. Int J Mol Sci. 2021;23:146. 35008569 10.3390/ijms23010146 PMC8745566

[160] Missiaen R Mazzone M Bergers G. The reciprocal function and regulation of tumor vessels and immune cells offers new therapeutic opportunities in cancer. Semin Cancer Biol. 2018;52(Pt 2):107–16. 29935312 10.1016/j.semcancer.2018.06.002 PMC6548870

[161] Li X Shang S Wu M Song Q Chen D. Gut microbial metabolites in lung cancer development and immunotherapy: novel insights into gut–lung axis. Cancer Lett. 2024;598:217096. 38969161 10.1016/j.canlet.2024.217096

[162] Paddock MN Field SJ Cantley LC. Treating cancer with phosphatidylinositol-3-kinase inhibitors: increasing efficacy and overcoming resistance. J Lipid Res. 2019;60:747–52. 30718284 10.1194/jlr.S092130 PMC6446698

[163] Markman B Dienstmann R Tabernero J. Targeting the PI3K/Akt/mTOR pathway--beyond rapalogs. Oncotarget. 2010;1:530–43. 21317449 10.18632/oncotarget.188 PMC3248125

[164] Noorolyai S Shajari N Baghbani E Sadreddini S Baradaran B. The relation between PI3K/AKT signalling pathway and cancer. Gene. 2019;698:120–8. 30849534 10.1016/j.gene.2019.02.076

[165] Hirsch E Lembo G Montrucchio G Rommel C Costa C Barberis L. Signaling through PI3Kgamma: a common platform for leukocyte, platelet and cardiovascular stress sensing. Thromb Haemost. 2006;95:29–35. 16543958

[166] Manning BD Toker A. AKT/PKB signaling: navigating the network. Cell. 2017;169:381–405. 28431241 10.1016/j.cell.2017.04.001 PMC5546324

[167] Li NN Meng XS Bao YR Wang S Li TJ. Evidence for the involvement of COX-2/VEGF and PTEN/Pl3K/AKT pathway the mechanism of Oroxin B treated liver cancer. Pharmacogn Mag. 2018;14:207–13. 29720833 10.4103/pm.pm_119_17 PMC5909317

[168] Sarbassov DD Guertin DA Ali SM Sabatini DM. Phosphorylation and regulation of Akt/PKB by the rictor-mTOR complex. Science. 2005;307:1098–101. 15718470 10.1126/science.1106148

[169] Saxton RA Sabatini DM. mTOR signaling in growth, metabolism, and disease. Cell. 2017;169:361–71. 10.1016/j.cell.2017.03.035 28388417

[170] Riely GJ Yu HA. EGFR: the paradigm of an oncogene-driven lung cancer. Clin Cancer Res. 2015;21:2221–6. 25979928 10.1158/1078-0432.CCR-14-3154 PMC4435716

[171] Zhang C Lan T Hou J. NOX4 promotes non-small cell lung cancer cell proliferation and metastasis through positive feedback regulation of PI3K/Akt signaling. Oncotarget. 2014;5:4392–405. 24946933 10.18632/oncotarget.2025 PMC4147332

[172] Zhang R Dong Y Sun M. Tumor-associated inflammatory microenvironment in non-small cell lung cancer: correlation with FGFR1 and TLR4 expression via PI3K/Akt pathway. J Cancer. 2019;10:1004–12. 30854106 10.7150/jca.26277 PMC6400805

[173] Lei Y Zhong C Zhang J. Senescent lung fibroblasts in idiopathic pulmonary fibrosis facilitate non-small cell lung cancer progression by secreting exosomal MMP1. Oncogene. 2025;44:769–81. 39663393 10.1038/s41388-024-03236-5 PMC11888990

[174] Dan HC Antonia RJ Baldwin AS. PI3K/Akt promotes feedforward mTORC2 activation through IKKα. Oncotarget. 2016;7:21064–75. 27027448 10.18632/oncotarget.8383 PMC5008269

[175] Lee MS Jeong MH Lee HW. PI3K/AKT activation induces PTEN ubiquitination and destabilization accelerating tumourigenesis. Nat Commun. 2015;6:7769. 26183061 10.1038/ncomms8769 PMC4518267

[176] Xu Z Wu S Tu J. RACGAP1 promotes lung cancer cell proliferation through the PI3K/AKT signaling pathway. Sci Rep. 2024;14:8694. 38622149 10.1038/s41598-024-58539-0 PMC11018837

[177] Li R Chai L Lei L Guo R Wen X. CDKL3 promotes non-small cell lung cancer by suppressing autophagy via activation of PI3K/Akt/mTOR pathway. Mol Biotechnol. 2023;65:1421–31. 36630073 10.1007/s12033-023-00656-8

[178] Yu F Tan W Chen Z. Nitidine chloride induces caspase 3/GSDME-dependent pyroptosis by inhibting PI3K/Akt pathway in lung cancer. Chin Med. 2022;17:115. 36175965 10.1186/s13020-022-00671-y PMC9524076

[179] Moghbeli M. PI3K/AKT pathway as a pivotal regulator of epithelial-mesenchymal transition in lung tumor cells. Cancer Cell Int. 2024;24:165. 38730433 10.1186/s12935-024-03357-7 PMC11084110

[180] Zhang Y Wang L Zhang M Jin M Bai C Wang X. Potential mechanism of interleukin-8 production from lung cancer cells: an involvement of EGF-EGFR-PI3K-Akt-Erk pathway. J Cell Physiol. 2012;227:35–43. 21412767 10.1002/jcp.22722

[181] Hanahan D Weinberg RA. Hallmarks of cancer: the next generation. Cell. 2011;144:646–74. 21376230 10.1016/j.cell.2011.02.013

[182] Yamamoto H Shigematsu H Nomura M. PIK3CA mutations and copy number gains in human lung cancers. Cancer Res. 2008;68:6913–21. 18757405 10.1158/0008-5472.CAN-07-5084 PMC2874836

[183] Balsara BR Pei J Mitsuuchi Y. Frequent activation of AKT in non-small cell lung carcinomas and preneoplastic bronchial lesions. Carcinogenesis. 2004;25:2053–9. 15240509 10.1093/carcin/bgh226

[184] Tan AC. Targeting the PI3K/Akt/mTOR pathway in non-small cell lung cancer (NSCLC). Thorac Cancer. 2020;11:511–8. 31989769 10.1111/1759-7714.13328 PMC7049515

[185] Dagogo-Jack I Valiev I Kotlov N. B-Cell infiltrate in the tumor microenvironment is associated with improved survival in resected lung adenocarcinoma. JTO Clin Res Rep. 2023;4:100527. 37521368 10.1016/j.jtocrr.2023.100527 PMC10372172

[186] Li S Zhou G Liu W Ye J Yuan F Zhang Z. Curcumol inhibits lung adenocarcinoma growth and metastasis via inactivation of PI3K/AKT and Wnt/-Catenin pathway. Oncol Res. 2021;28:685–700. 32886059 10.3727/096504020X15917007265498 PMC8420902

[187] Zhao B Hui X Jiao L. A TCM formula YYWY inhibits tumor growth in non-small cell lung cancer and enhances immune-response through facilitating the maturation of dendritic cells. Front Pharmacol. 2020;11:798. 32595493 10.3389/fphar.2020.00798 PMC7301756

[188] Cai F Chen M Zha D. Curcumol potentiates celecoxib-induced growth inhibition and apoptosis in human non-small cell lung cancer. Oncotarget. 2017;8:115526–45. 29383179 10.18632/oncotarget.23308 PMC5777791

[189] Fang X Zhu Y Zhang T. Fucoxanthin inactivates the PI3K/Akt signaling pathway to mediate malignant biological behaviors of non-small cell lung cancer. Nutr Cancer. 2022;74:3747–60. 35838029 10.1080/01635581.2022.2091149

[190] Ming JX Wang ZC Huang Y. Fucoxanthin extracted from Laminaria Japonica inhibits metastasis and enhances the sensitivity of lung cancer to Gefitinib. J Ethnopharmacol. 2021;265:113302. 32860893 10.1016/j.jep.2020.113302

[191] Zhou B Yang Y Pang X Shi J Jiang T Zheng X. Quercetin inhibits DNA damage responses to induce apoptosis via SIRT5/PI3K/AKT pathway in non-small cell lung cancer. Biomed Pharmacother. 2023;165:115071. 37390710 10.1016/j.biopha.2023.115071

[192] Ge Z Xu M Ge Y. Inhibiting G6PD by quercetin promotes degradation of EGFR T790M mutation. Cell Rep. 2023;42:113417. 37950872 10.1016/j.celrep.2023.113417

[193] Jiang M Zhou LY Xu N An Q. Hydroxysafflor yellow A inhibited lipopolysaccharide-induced non-small cell lung cancer cell proliferation, migration, and invasion by suppressing the PI3K/AKT/mTOR and ERK/MAPK signaling pathways. Thorac Cancer. 2019;10:1319–33. 31055884 10.1111/1759-7714.13019 PMC6558494

[194] Wang CH Li XF Jin LF Zhao Y Zhu GJ Shen WZ. Dieckol inhibits non-small-cell lung cancer cell proliferation and migration by regulating the PI3K/AKT signaling pathway. J Biochem Mol Toxicol. 2019;33:e22346. 31291034 10.1002/jbt.22346 PMC6771741

[195] Yu J Zhang L Peng J. Dictamnine, a novel c-Met inhibitor, suppresses the proliferation of lung cancer cells by downregulating the PI3K/AKT/mTOR and MAPK signaling pathways. Biochem Pharmacol. 2022;195:114864. 34861243 10.1016/j.bcp.2021.114864

[196] Hsieh YS Liao CH Chen WS Pai JT Weng MS. Shikonin inhibited migration and invasion of human lung cancer cells via suppression of c-met-mediated epithelial-to-mesenchymal transition. J Cell Biochem. 2017;118:4639–51. 28485480 10.1002/jcb.26128

[197] Niu H Wang D Wen T. Anwuligan inhibits the progression of non-small cell lung cancer via let-7c-3p/PI3K/AKT/mTOR axis. Cancer Med. 2023;12:5908–25. 36412203 10.1002/cam4.5382 PMC10028152

[198] Zhu F Ren Z. Icariin inhibits the malignant progression of lung cancer by affecting the PI3K/Akt pathway through the miR‑205‑5p/PTEN axis. Oncol Rep. 2022;47:115. 35514319 10.3892/or.2022.8326 PMC9100476

[199] Yang M Wang H Zhou M. The natural compound sulforaphene, as a novel anticancer reagent, targeting PI3K-AKT signaling pathway in lung cancer. Oncotarget. 2016;7:76656–66. 27765931 10.18632/oncotarget.12307 PMC5363538

[200] Li YC He SM He ZX. Plumbagin induces apoptotic and autophagic cell death through inhibition of the PI3K/Akt/mTOR pathway in human non-small cell lung cancer cells. Cancer Lett. 2014;344:239–59. 24280585 10.1016/j.canlet.2013.11.001

[201] Zhong J Hua Y Zou S Wang B. Juglone triggers apoptosis of non-small cell lung cancer through the reactive oxygen species -mediated PI3K/Akt pathway. PLoS One. 2024;19:e0299921. 38814975 10.1371/journal.pone.0299921 PMC11139338

[202] Li W Li C Ma L Jin F. Resveratrol inhibits viability and induces apoptosis in the small‑cell lung cancer H446 cell line via the PI3K/Akt/c‑Myc pathway. Oncol Rep. 2020;44:1821–30. 32901891 10.3892/or.2020.7747 PMC7550979

[203] Hu S Li X Xu R. The synergistic effect of resveratrol in combination with cisplatin on apoptosis via modulating autophagy in A549 cells. Acta Biochim Biophys Sin (Shanghai). 2016;48:528–35. 27084520 10.1093/abbs/gmw026 PMC4913517

[204] Gu S Chen C Jiang X Zhang Z. Resveratrol synergistically triggers apoptotic cell death with arsenic trioxide via oxidative stress in human lung adenocarcinoma A549 cells. Biol Trace Elem Res. 2015;163:112–23. 25431299 10.1007/s12011-014-0186-2

[205] Lu T Wang Y Liu F. Synergistic inhibitory effect of berberine and low-temperature plasma on non-small-cell lung cancer cells via PI3K–AKT-driven signaling axis. Molecules. 2023;28:7797. 38067530 10.3390/molecules28237797 PMC10708101

[206] Liao W Zhang R Chen G. Berberine synergises with ferroptosis inducer sensitizing NSCLC to ferroptosis in p53-dependent SLC7A11-GPX4 pathway. Biomed Pharmacother. 2024;176:116832. 38850659 10.1016/j.biopha.2024.116832

[207] Liu X Jiang Q Liu H Luo S. Vitexin induces apoptosis through mitochondrial pathway and PI3K/Akt/mTOR signaling in human non-small cell lung cancer A549 cells. Biol Res. 2019;52:7. 30797236 10.1186/s40659-019-0214-y PMC6387544

[208] Zhou X Zhou R Li Q. Cardamonin inhibits the proliferation and metastasis of non-small-cell lung cancer cells by suppressing the PI3K/Akt/mTOR pathway. Anticancer Drugs. 2019;30:241–50. 30640793 10.1097/CAD.0000000000000709

[209] Provencio M Nadal E Insa A. Neoadjuvant chemotherapy and nivolumab in resectable non-small-cell lung cancer (NADIM): an open-label, multicentre, single-arm, phase 2 trial. Lancet Oncol. 2020;21:1413–22. 32979984 10.1016/S1470-2045(20)30453-8

[210] Mohiuddin M Kasahara K. Paclitaxel impedes EGFR-mutated PC9 Cell growth via reactive oxygen species-mediated DNA damage and EGFR/PI3K/AKT/mTOR signaling pathway suppression. Cancer Genomics Proteomics. 2021;18:645–59. 34479917 10.21873/cgp.20287 PMC8441765

[211] Li Z Shen Y Xin J. Cryptotanshinone alleviates radiation-induced lung fibrosis via modulation of gut microbiota and bile acid metabolism. Phytother Res. 2023;37:4557–71. 37427974 10.1002/ptr.7926

[212] Lindeman NI Cagle PT Aisner DL. Updated molecular testing guideline for the selection of lung cancer patients for treatment with targeted tyrosine kinase inhibitors: guideline from the College of American Pathologists, the International Association for the Study of Lung Cancer, and the Association for Molecular Pathology. J Mol Diagn. 2018;20:129–59. 29398453 10.1016/j.jmoldx.2017.11.004

